# Reliable fluorescence technique to detect the antibiotic colistin, a possible environmental threat due to its overuse

**DOI:** 10.1038/s41598-022-13471-z

**Published:** 2022-06-03

**Authors:** Saurodeep Mandal, Arpan Dey Bhowmik, Alpana Mukhuty, Shampa Kundu, Khai-Nghi Truong, Kari Rissanen, Ansuman Chattopadhyay, Prithidipa Sahoo

**Affiliations:** 1 Department of Chemistry, Visva-Bharati, Santiniketan, West Bengal 731235 India; 2 Department of Zoology, Visva-Bharati, Santiniketan, West Bengal 731235 India; 3 Department of Chemistry, University of Jyvaskyla, Survontie 9 B, P.O. Box 35, 40014 Jyvaskyla, Finland

**Keywords:** Environmental sciences, Chemistry

## Abstract

Colistin, considered a drug of last resort as it is effective towards multidrug-resistant Gram-negative bacterial infections. Oral administration of colistin in the poultry industry is a common practice, not only to prevent and reduce bacterial infections, but also as a rapid-growth promoter. Long-term exposure to any antibiotic will eventually lead to the development of bacterial resistance towards all antibiotics through various mechanisms in the physiological system and environment. Chicken is the most consumed source of animal protein for humans throughout the world. In addition, the manure of poultry, containing traces of the used antibiotics, is being used in farming. Exposure to excess amounts of colistin causes a great concern not only to the humans but to the environment as a whole. In the present contribution, colistin has been detected in chicken hepatocyte cells through in vivo confocal microscopy. In addition, the amount of colistin in the chicken excrements has been estimated. A simple chemosensor NAF, a dye-based on napthaldehyde furfural, was developed for the detection of colistin, supplemented with experimental evidence and theoretical calculations.

## Introduction

Ever since the discovery of penicillin, both practitioners and common people have relied upon the wide use of antibiotics. This has resulted in the greatest global health threat of the twenty-first century: namely, the development of antibiotic resistance among bacteria^1,2^. We have, consequently, an insufficiency^3^ of new generation antibiotics in our reserve list which are acquiescent to different bacterial species. A few among these antibiotics, such as polymyxins, are effective against topical Gram-negative infections. Colistin, commonly known as polymyxin E, belongs to an old class of antibiotics discovered in the year of 1947; it acts by disrupting the bacterial membranes resulting in cellular death^4^. Colistin and colistimethate sodium have been approved for medical purposes in the USA since 1970 and found to be effective in treating infections caused by *Escherichia*, *Pseudomonas,* and *Klebsiella* species^5^. Colistin is considered a drug of last resort to be potent towards multidrug-resistant Gram-negative bacterial infections including pneumonia^6^. It has found a place in the essential medicine list by the World Health Organisation and has also been categorised as a critically important human medicine^7,8^. Literature data suggest that the rapid spread of colistin resistance among bacterial strains occurs through horizontally transmissible mobilized colistin resistance (*mcr*) carrying plasmids^9^. *Escherichia coli* (*E. coli*) lineages having colistin-resistant gene *mcr-1* were isolated from domestic animals and humans living in the same household in Ecuador*.* The results suggested that *mcr-1* gene is horizontally transferred amongst *E. coli* lineages in humans from the domestic animal in the household^10^. Recently, colistin resistant *mcr-4* and *mcr-5* genes were isolated from the upper and lower alimentary tract of poultry chicken and pigs in China^11^. Furthermore, *mcr-1* carrying Enterobacteriaceae was found in the surrounding environment of a commercial poultry farm^12^. The manure of poultry animals has very widespread use in organic farming nowadays. Carbapenemase-producing Enterobacteriaceae *Klebsiella pneumoniae* that are highly colistin-resistant, were identified from fresh raw vegetables in Puducherry market, India^13^ and from the isolates of hospitalised patients in London^14^. Although the governments of a few countries banned the production, distribution, and sale of colistin and its formulation for food-producing animal poultry, aquafarming, and animal feed, colistin is being sold in the open market to date and is aggressively used in different poultry farms. Oral administration of colistin in chicken is a common practice in the poultry industry. It prevents and reduces the risk of bacterial infections, besides acting as a rapid growth promoter. Consequently, we have chosen the chemosensing pathway^15^ to study the accumulation pattern of colistin in the physiological system of the broiler poultry chicken. Thus, we have synthesised a napthaldehyde furfural based chemosensor, **NAF** for the in vivo and in vitro detection of colistin in hepatocyte cells of the animal system through fluorometric sensing. **NAF** has been synthesised and well-characterised by NMR, HRMS fluorometric experiments, and X-ray diffraction analysis (Fig. 6, Supplementary Figs. S1–S4).

## Results and discussion

Several experiments have been conducted to investigate the binding mechanism of colistin with the probe **NAF**, such as absorbance, fluorescence titration, ^1^H NMR titration, and computational studies. Absorption spectra of **NAF** has depicted two major absorption bands at 231 nm and 384 nm in acetonitrile–water (2:5, v/v), buffered with 10 mM PBS buffer (pH 7.0). The emission intensity of **NAF** is enhanced significantly with the emission maxima at 504 nm (λ_ex_ = 384 nm) upon incremental addition of colistin. The luminescence intensity is increased rapidly by fourfolds with the turn on fluorescence of the probe **NAF** from non-fluorescent to bright green colour; this is consistent with the emission peak at 504 nm (Supplementary Fig. S5). **NAF** possesses high association constant value, i.e., 4.638 × 10^5^ M^−1^ for colistin (Supplementary Fig. S6), derived from Benesi-Hildebrand equation^16^. The limit of detection (LOD) and limit of quantification for probe **NAF** towards colistin is found to be 1.576 × 10^–8^ M and 5.253 × 10^–8^ M, respectively, estimated from the linear relationship between concentration of colistin and fluorescence intensity (Supplementary Fig. S7, Table S1). The binding stoichiometry, obtained from Job’s plot, reveals 1:1 stoichiometric interaction between **NAF** and colistin (Supplementary Fig. S8).

Selectivity studies have also been conducted with **NAF** in presence of different metal ions, relevant biomolecules, vitamins, and other antibiotics. They do not interfere with, or exhibit any apparent changes in, the fluorescence response of colistin with **NAF** (Supplementary Fig. S9).

The pH titration curve has shown that the probe NAF remains unaltered within the pH range of 4 to 8, but fluorescence intensity increases moderately with increasing basicity (above pH 10, Supplementary Fig. S10). This feature helps to obtain the practical utility of **NAF** as well as in the real time biological applications.

Ground-state optimised structures of **NAF** and **NAF-**colistin complex have been studied using hybrid exchange–correlation functional B3LYP (Becke, three parameters, Lee–Yang–Parr) and 6-31G (d,p) basis set implemented at Gaussian09 program^17^. CPCM (Conductor like Polarizable Continuum Model) solvent model is incorporated to consider the solvent (acetonitrile) effect. During DFT calculations, it is observed that **NAF** is more energetically favoured, but while interacting with colistin it changes some of its structural conformation in order to interact strongly with colistin-minimising the energy significantly, hence giving a stable conjugated complex.

**Figure 1:**
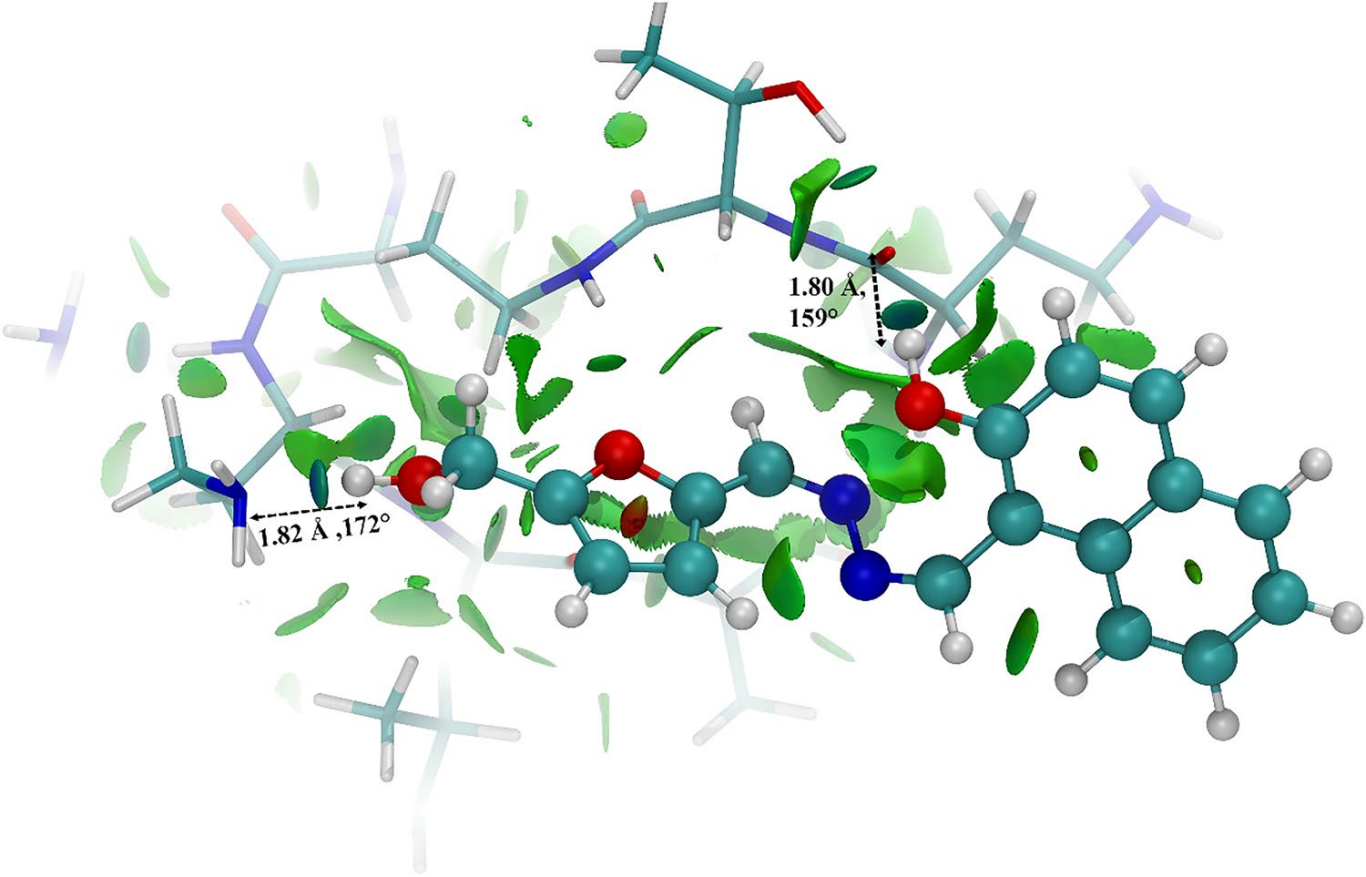
NCI isosurface for the **NAF**-colistin complex obtained from the non-covalent interactions, where RDG cutoff is 0.5 coloured over − 0.1 sign(λ_2_)ρ + 0.1 a.u. Sphere colour code representation: cyan = carbon, white = hydrogen, yellow = sulfur, blue = nitrogen, red = oxygen. The bond lengths (Å) and bond angles (°) are described in respective panel.

The electronic transition from S_0_ → S_1_ and S_0_ → S_11_ corresponds to the termination of excited state intramolecular proton transfer (ESIPT) process in the complex, present in **NAF** previously (Supplementary Figs. S11, S12, S13, S14 and Tables S2, S3). The NCI plot states that multiple interaction peaks have appeared at the low-density low gradient region of "s" vs "sin(λ_2_)ρ" plot (Supplementary Fig. S15) signifying several non-covalent interactions. Moreover, considering the spatiotemporal as well as structural orientations, bond distances and bond angles are consistent with the strong hydrogen bonding interactions between **NAF** and colistin (Fig. 1).

The ^1^H NMR titration in DMSO-*d*_*6*_ was carried out to explore the interaction mechanism in-between **NAF** and colistin. In PMR titration, the singlet proton peaks of phenolic OH and the aliphatic OH in **NAF** at 13.07 ppm and 5.48 ppm, respectively, completely disappear upon gradual addition of colistin. This observation indicates the formation of strong hydrogen bonding interaction during complexation (Supplementary Fig. S16).

**Figure 2:**
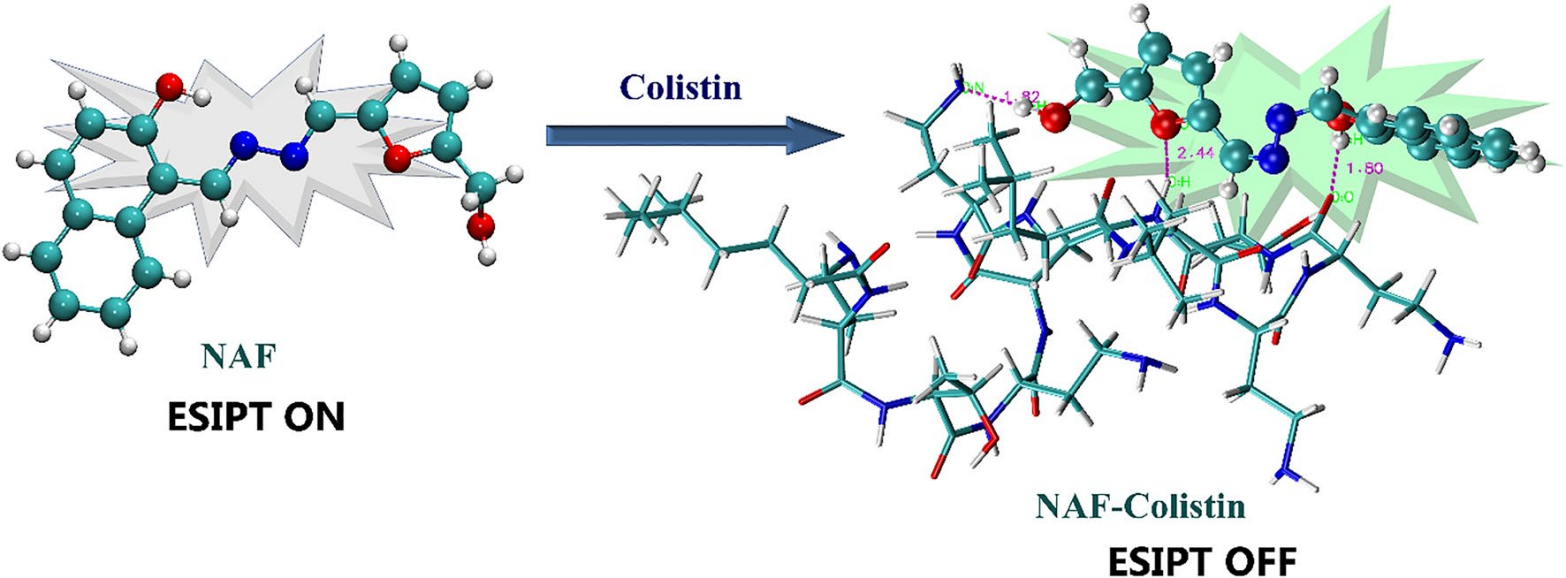
Binding mechanism of NAF with colistin.

Experimental findings and theoretical investigations led us to postulate a plausible binding mechanism of **NAF** with colistin represented in Fig. 2. According to our proposed mechanism **NAF** can take part in the tautomerisation process due to its favourable optimised enol structure acquired from crystallographic data, facilitates the excited state intramolecular proton transfer (ESIPT) mechanism responsible for the non-fluorescence nature of the free sensor. The two different emission bands at 350 nm and 504 nm responsible for normal emission and phototautomer emission, along with a large stokes shift strongly ensure for ESIPT mechanism. The hydroxyl proton from naphthalene moiety of NAF, directly responsible for the ESIPT mechanism, forms strong hydrogen bonding interaction (confirmed by NMR titration) with colistin and the tautomerisation process gets inhibited accordingly. The suppression of ESIPT mechanism initiates strong green, fluorescent colour of NAF-colistin complex.

Further, **NAF** has been used potentially to establish its practical applicability to detect colistin accumulation or retention possibility in a poultry chicken's physiological systems and colistin contamination in the environment through their excretion.

**Figure 3:**
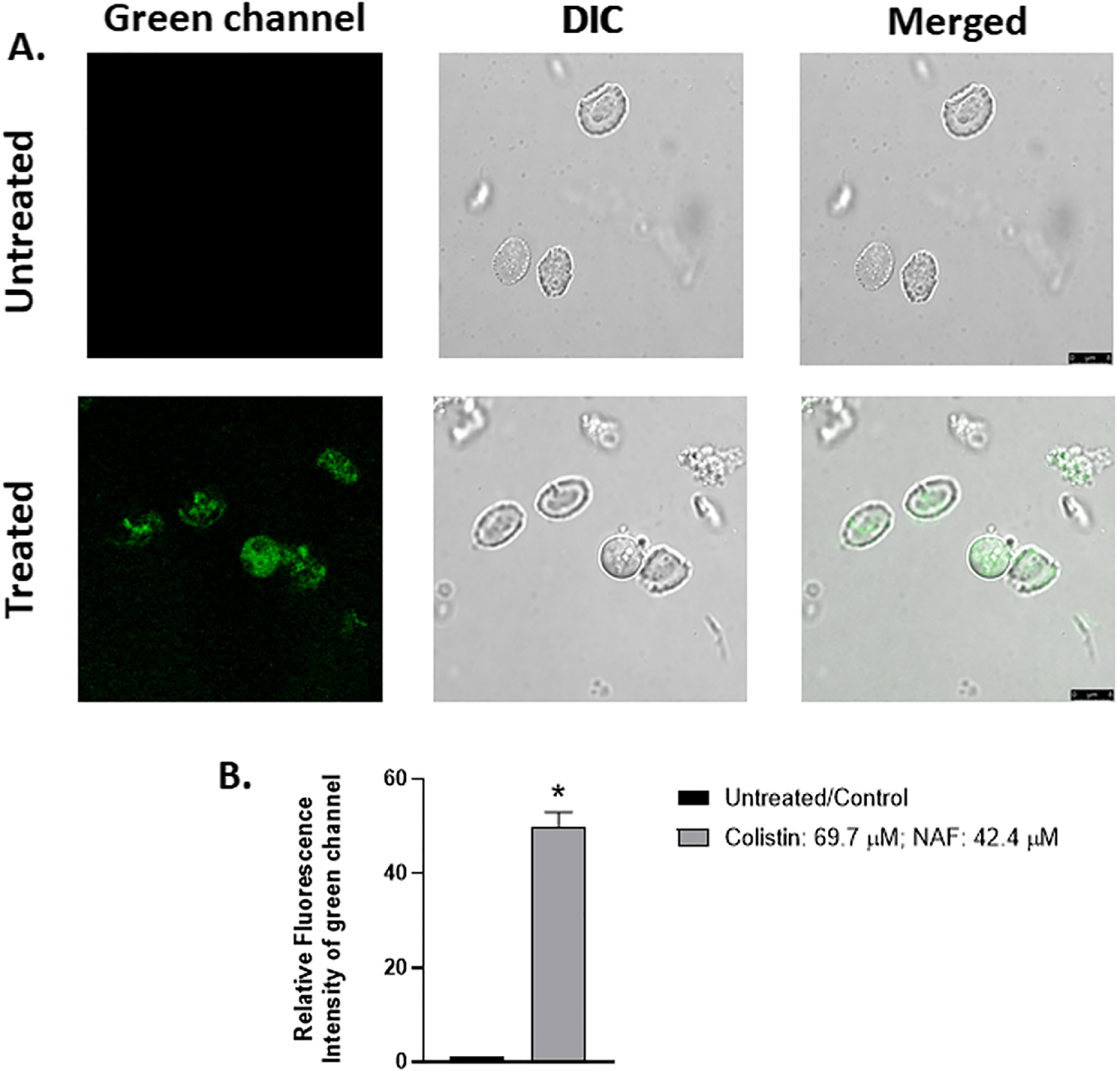
Confocal microscopic images of isolated hepatocytes from zebrafish (**A**) that were either left untreated (control) 42.4 µM of NAF for 45 min or treated with 69.7 µM of colistin and 42.4 µM of NAF for 75 and 45 min, respectively. Green channel, DIC images and merged images of untreated and treated cells are shown in consecutive rows. Scale bar is 8 μm. Zoom factor = 3.5. Magnification = ×630. (**B**) Relative fluorescence intensity of the above-mentioned images was quantified using ImageJ v 1.46 software and graphed.

An in vivo experiment was performed in chicken’s hepatocyte cells to execute the colistin accumulation. We decided to proceed with the liver system, more specifically hepatocyte cell, which receives a substantial amount of nutrients and harmful compounds through the digestive tract and portal vein^18^. Hepatocytes are considered crucial as they comprise 80% of the liver mass^19^ and perform various functions, e.g. protein secretion, cholesterol uptake, glycogen storage, immune surveillance, and detoxification of drug, alcohol, chemicals^20^. Before actually working in vivo*,* we first focused on in vitro experiments to check the possibility of involving our detection procedure in biological systems. We started with simple zebrafish model due to its more than 70% orthologous genetic similarity with humans^21^, its small size and its rapid growth (Supplementary Table S4). First, by applying optimised conditions, confocal microscopic images were obtained; this was taken as evidence that both colistin and NAF possess the capability to penetrate cell membrane. Fluorescence signal is obtained upon interaction of the probe and analyte inside the cell, more specifically in the cytoplasmic region (Fig. 3). We have conducted both the time-dependent kinetic studies and dose-dependent kinetic experiments in order to optimise the most favoured conditions (Supplementary Figs. S17 and S18). The optimised dose for colistin (69.7 µM) and **NAF** (42.4 µM) were selected due to the significantly high intensity of green fluorescence. Similarly, the incubation time for colistin and **NAF** was optimised at 75 and 45 min, respectively.

**Figure 4:**
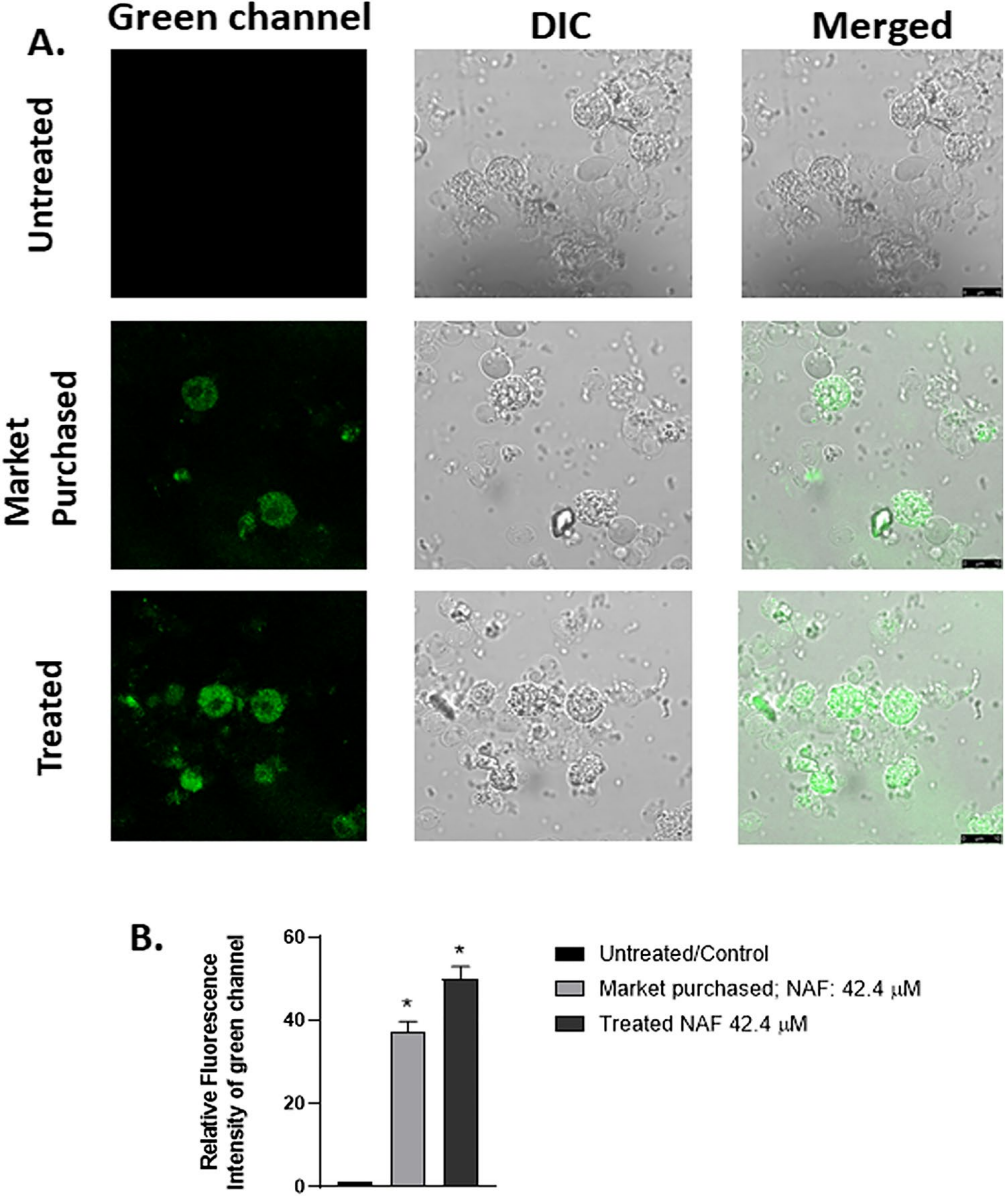
Confocal microscopic images of isolated hepatocytes from chicken (**A**) that were either left untreated (control) or treated with Colistin for 32 days (400 mg/day first 15 days, 600 mg/day for the rest of the treatment period) and the purchased one with 42.4 µM of **NAF** for 45 min. Green channel, DIC images and merged images untreated, purchased and treated cells are shown in consecutive rows. Scale bar is 10 μm. Zoom factor = 2.4. Magnification = ×630. (**B**) Relative fluorescence intensity of the above-mentioned images was quantified using ImageJ v 1.46 software and graphed.

After obtaining convincing results from the zebrafish model, we further moved to accomplish in vivo experiment in chicken hepatocyte cells. A set of chicken (n = 5) were supplemented with colistin (dosage 400 mg for first 15 days and 600 mg/day before dissection, dosage mentioned here administered orally with food, it is not the actual amount of intake^22^) in food and drinking water for prolonged period (32 days, Supplementary Table S5). In keeping with the previous in vitro experiments, we have restricted ourselves to in vivo dose-dependent kinetic studies (Supplementary Fig. S19), maintaining the incubation time the same as previously optimised. The optimised dose for **NAF** was found to be 42.4 µM. Confocal microscopic images of in vivo experiments ensure the accumulation of colistin in the hepatocytes isolated from the liver of poultry chicken. Chicken treated with colistin for prolonged period and chicken purchased from the market significantly depict the presence of colistin in the cells (Fig. 4). Green fluorescence is observed in the cytoplasmic region of the hepatocyte cells.

**Figure 5:**
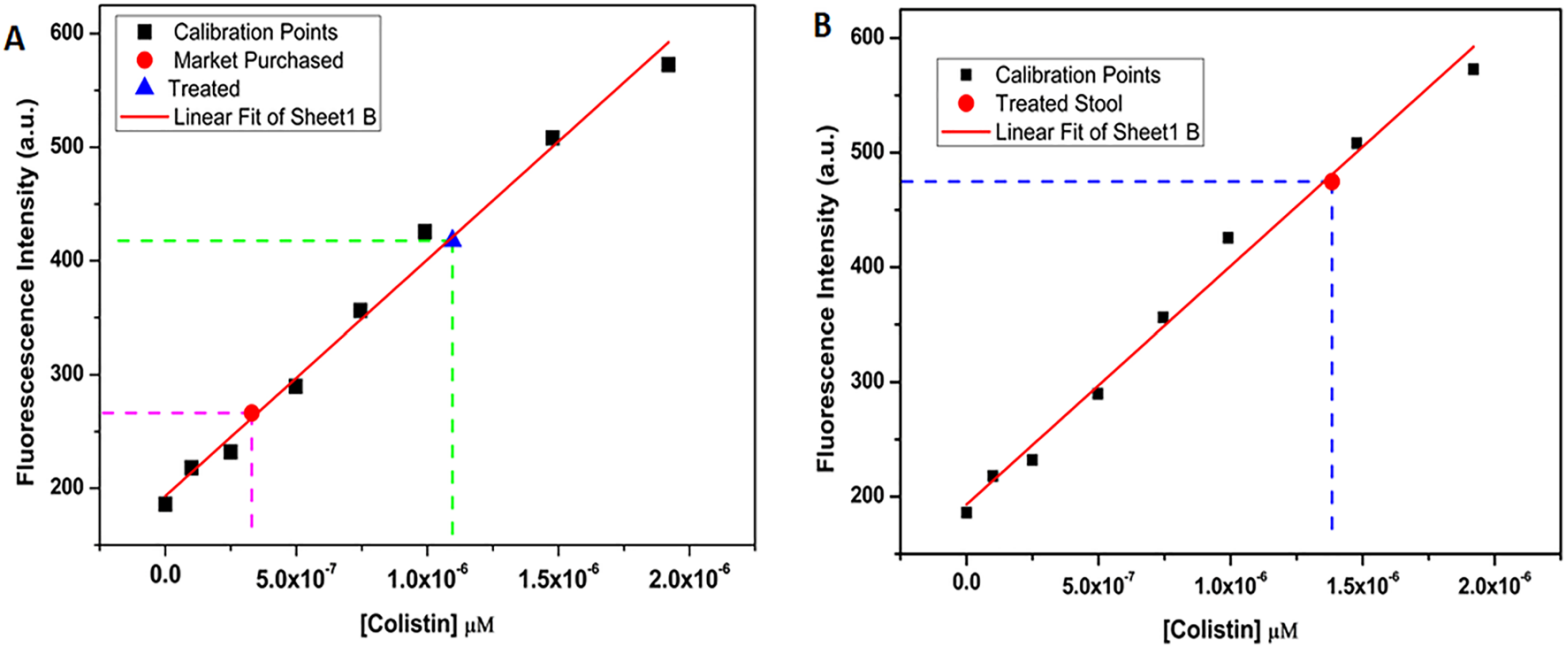
Calibration curve: (**A**) concentration of colistin obtained from hepatocyte cell lysate of market purchased chicken (red), colistin treated chicken (blue) and (**B**) concentration of colistin from the manure (stool and urine) of treated chicken.

**Figure 6:**
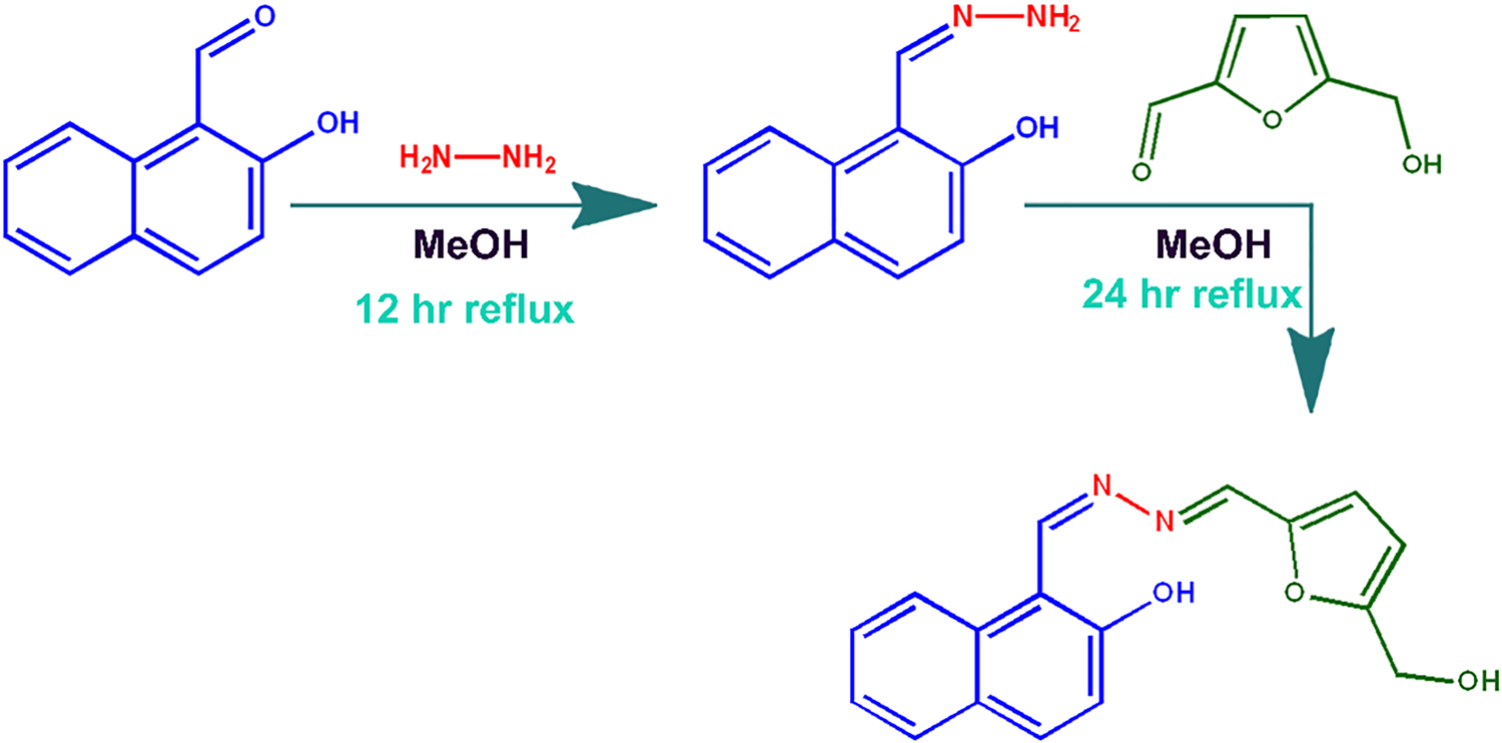
Synthesis of the probe **NAF**.

Furthermore, the amount of colistin was estimated in the extract of liver hepatocyte cells and the excreted manure (stool and urine) sample collected from the chicken samples. Incidentally, the results demonstrate that the detectable amount of residual colistin was present both in hepatocyte cell lysate and in the manure excreted by the chicken. The concentration obtained is represented successfully by comparing with standard fluorescence curve such as 0.32 µM, 1.17 µM and 1.38 µM for poultry chicken, colistin treated chicken and from stool of treated chicken, respectively (Figs. 5, 6).

## Conclusions

The regular and long-term administration of colistin has deleterious effects on public and environmental health. A turned-on fluorescence technique has been used to recognise colistin in hepatocyte cells of poultry chicken by using a novel naphthalene-based simple luminescent probe (**NAF**) in neutral pH. The binding mechanism of **NAF** with colistin has been well recognised by spectroscopic and computational studies. The potentiality of **NAF** has been extended by representing in vitro and in vivo imaging of hepatocytes cells of zebrafish and chicken, respectively. The accumulation of colistin in the liver and residual retention in the excrements of the chicken has been estimated using the fluorescence intensity of **NAF**. Nowadays, poultry manures are being used as fertilisers for organic farming, so colistin may get deposited eventually in land and water bodies, and can be a great threat to the environment in the near future. Antibiotic residues are often detected in waste water; thus, discharge of manufacturing industries, and of animal husbandry is a potential threat to the agro-ecosystem, aquatic and land animals and alteration of human microbiome that leads to human antibiotic resistance. So, it is high time people became conscious that the uninterrupted use of such antibiotics is indirectly detrimental to our health and eventually enhances multidrug resistance towards Gram-negative bacterial infections.

## Methods

### Synthesis of NAF

**NAF** was synthesised from compound A by single-step imine formation reaction. Compound A (1.5 g, 8 mmol) and methanol (40 ml) were placed in a 100 mL round-bottom flask. After 5 min of stirring, 5-hydroxymethyl furfuldehyde (1 mL, 9.6 mmol) was added dropwise and refluxed for 24 h at 83 °C. On completion of the reaction (monitored by TLC), the solvent was evaporated completely under reduced vapour pressure, then extracted with chloroform and water. After drying it over anhydrous Na_2_SO_4_, the organic layer was evaporated completely. The residue was purified by column chromatography with the eluent CHCl_3_:EtOAc (6:1, v/v) in order to obtain the pure product **NAF** with 87% yield.

A stock solution of NAF (10 μM) was prepared in acetonitrile–water. Colistin solutions of different concentrations were prepared in Millipore water. All the experiments have been carried out in acetonitrile–water (2:5, v/v), buffered with (10 mM phosphate buffer, pH 7.0). During the titration, each time a 10 μM solution of NAF was filled in quartz optical cell of 1 cm optical path length and colistin solution was added incrementally into the quartz optical cell using a micropipette. Spectral data have been recorded immediately after the addition of colistin solution (filter open, slit 10/10). Fluorescence spectra were recorded on a Perkin Elmer Model LS 55 spectrophotometer. UV spectra have been recorded on a SHIMADZU UV-3101PC spectrophotometer.

### Crystallographic data

Single crystal X-ray data of **NAF** was measured using a dual-source Rigaku Super Nova diffractometer equipped with an Atlas detector and an Oxford Cryostream cooling system using mirror-monochromated Cu-K_α_ radiation (λ = 1.54184 Å). Data collection and reduction were performed using the program *CrysAlisPro* and Gaussian face-index absorption correction method was applied^23^. The structure was solved with Direct Methods (*SHELXS*)^24^ and refined by full-matrix least-squares based on *F*^*2*^ using *SHELXL*-2015^24–26^. Non-hydrogen atoms were assigned anisotropic displacement parameters unless stated otherwise. The hydrogen atoms bonded to oxygen were located from Fourier difference maps and refined with an O–H distance restraint of approximately 0.92 Å. Other hydrogen atoms were placed in idealised positions and included as riding. Isotropic displacement parameters for all H atoms were constrained to multiples of the equivalent displacement parameters of their parent atoms with U_iso_(H) = 1.2 U_eq_(parent atom). A few reflections with large discrepancies between the calculated and observed structure factors have been omitted from the least-squares refinement as outliers. The crystal of **NAF** proved to be a non-merohedral twin. In the twin refinement, an approximately modified set of intensity data taking the partially overlapped diffraction of a two-component twin into account (HKLF5 format in SHELXL-15) gave significantly improved convergence results and relative domain fractions of 0.562 and 0.438 (before twin refinement: R_1_[F^2^ > 2σ(F^2^)] = 0.1686, wR_2_(F^2^) = 0.4561, GooF = 2.982, ∆ρ_max_/∆ρ_min_ (eÅ^–3^) = 0.907/− 0.508). The single crystal X-ray data, experimental details as well as CCDC number are given below.

Crystal data for **NAF** (obtained via solvent diffusion from MeCN/petroleum benzene at ambient temperature): CCDC-2123611, C_17_H_14_N_2_O_3_, M = 294.30 gmol^–1^, yellow rod, 0.33 × 0.09 × 0.09 mm^3^, monoclinic, space group *P*2_1_/*c* (No. 14), a = 13.5692(9) Å, b = 4.4495(3) Å, c = 24.0801(14) Å, α = 90°, β = 99.985(2)°, γ = 90°, V = 1431.84(16) Å^3^, Z = 4, D_calc_ = 1.365 gcm^–3^, F(000) = 616, µ = 0.781 mm^–1^, T = 120(2) K, θ_max_ = 73.95°, 4918 total reflections, 1774 with I_o_ > 2σ(I_o_), R_int_ = 0.0377, 2717 data, 206 parameters, 2 restraints, GooF = 1.099, R_1_ = 0.0994 and wR_2_ = 0.2773 [I_o_ > 2σ(I_o_)], R_1_ = 0.1251 and wR_2_ = 0.2873 (all reflections), 0.420 < d∆ρ < − 0.368 eÅ^–3^.

### In vitro experiment with zebrafish

#### Maintenance of zebrafish

Adult wild-type zebrafish were purchased and kept in aquariums (water temperature: 25 ± 1 °C). Commercial fish food (Tetrabits, Germany) was used as a feed two times a day. A photoperiod of 14 h:10 h light:dark cycle was maintained. All animal experiments were carried out according to the guidelines of Institutional Animal Ethics Committee of Visva-Bharati University (1819/GO/Re/S/15/CPCSEA dated 01.09.2015) and approved by the committee according to Indian law. For isolation of zebrafish hepatocyte, fish (N = 20) were euthanised and livers were dissected out. Then the tissues were washed in sterile PBS and collected in Leibovitz L-15 media. After collagenase treatment at 37 °C, the mass was passed through sterile nylon mesh and centrifuged at 2000 rpm × 2 min. Cell pellet was re-suspended in the same media and kept for 30 min at 28 °C incubator; meanwhile we checked the cell viability. Cell density was measured by hemocytometer in both the cases.

Isolated hepatocytes from zebrafish cells were treated with different concentrations of colistin (0, 27, 69.7, 91.1 µM and **NAF** 12.1, 42.4 49.3 µM) for 75 and 45 min respectively. In another experiment, isolated hepatocytes from zebrafish cells were treated with 69.7 µM and 42.4 µM colistin and **NAF** for different time (0, 45 and 30, 75 and 45, 180 and 120 min). The cells were then washed with PBS buffer (3 times) and mounted with PBS and immediately used for imaging under confocal laser scanning microscope [Leica TCS SP8]. The control or untreated cell samples were incubated with **NAF**. Green channel filter excitation was set at 450–490 nm, and emission at 515–565 nm. Fluorescence intensity of the images were quantified by ImageJ software and plotted respectively.

### In vivo experiment with poultry chicken

Two sets of chicken were kept for 36 days until the dissection took place and confocal microscopic images were obtained. From day 5, one set (n = 5) of poultry chicken was kept as control and the other set (n = 5) of chickens were treated (dosage 400 mg for first 15 days and 600 mg/day before dissection, dosage mentioned here administered orally with food, it is not the actual amount of intake)^24^ with colistin in food. Four times a day they were supplemented with commercially available food along with colistin (Purchased from local market as colistin sulfate). At the end of the treatment period (32 days) the chickens were sacrificed with ketamine anesthesia. Liver was perfused with the help of calcium-free perfusion buffer having EDTA and Hank’s solution. The liver was then dissected out, placed on a petri dish and perfused with the help of HEPES buffer containing 0.08% collagenase for 15 min. Liver pieces were then passed through sterile nylon mesh with complete Leibovitz-15 media. The hepatocytes were isolated by centrifugation at 50*g* × 2 min, washed 3 times with the Leibovitz-15 media and kept in incubator at 37 °C for 30 min; meanwhile we checked the cell viability.

Isolated hepatocytes from every group were then mounted and immediately used for imaging under confocal laser scanning microscope [Leica TCS SP8]. The control or untreated cell samples were incubated with **NAF**. Green channel filter excitation was set at 450–490 nm, and emission at 515–565 nm. Fluorescence intensity of the images were quantified by ImageJ software and plotted respectively.

### Preparation of cell lysate

Liver tissue was taken 0.8193 gm (market purchased) and 0.8226 g (colistin treated) in mass and extracted in aquas media by grinding and dissolving it in 8 mL chilled water followed by sonication for 3 min. After sonication filtration was done using whatman 42 filter paper. The filtrate was collected and centrifuged for 6 min, three times at 12,500 rpm at 9-degree centigrade. The supernatant was collected and used for fluorescence titration to obtain fluorescence emission data which was later utilised in plotting upon standard fluorescence titration curve and estimation of colistin present in the sample was checked.

### Preparation of manure extract

0.6037 g of excreted manure (stool and urine mixture) was dissolved in 4 mL of distilled water followed by centrifugation at 2000 rpm for 3 min and the supernatant was collected to perform the fluorescence titration experiments. The presence of residual colistin from the manure of the poultry chicken was estimated by plotting on the standard fluorescence titration curve.

### Statistical analysis

Each experiment was performed in triplicate and repeated for five times at least. Data are presented in mean ± S.D. and differences among the data were determined by Student's t-test and/or One-way ANOVA using Graph pad Prism 5.0. p < 0.05 was considered as statistically significant.

### Ethics declarations

We confirm that the present study is reported in accordance with ARRIVE guidelines. All animal experiments were carried out according to the guidelines of Institutional Animal Ethics Committee of Visva-Bharati University (1819/GO/Re/S/15/CPCSEA dated 01.09.2015).

## Supplementary Information

Supplementary Information.

## Data Availability

All data supporting this study and its findings are available within the article and its Supplementary Information or from the corresponding authors upon request. Crystallographic data has been deposited at the Cambridge Crystallographic Database Centre and is available on request (http://www.ccdc.cam.ac.uk) (Accession no CCDC-2123611).
