# Supplementary material for: Reliable fluorescence technique to detect the antibiotic colistin, a possible environmental threat due to its overuse
Source: Sci Rep. 2022 Jun 3;12:9307. doi: 10.1038/s41598-022-13471-z (PMC9166777; doi:10.1038/s41598-022-13471-z)
Supplement: Supplementary file 1 — Supplementary Information. [file 41598_2022_13471_MOESM1_ESM.docx]

**Electronic Supplementary Information**

**Reliable fluorescence technique to detect the antibiotic colistin, a possible environmental threat due to its overuse**

Saurodeep Mandal^1^, Arpan Dey Bhowmik^2^, Alpana Mukhuty^2^, Shampa Kundu^1^, Khai-Nghi Truong^3^, Kari Rissanen^3^,  Ansuman Chattopadhyay^2^ and Prithidipa Sahoo^1,*^

Corresponding Author Email: prithidipa@hotmail.com

^1^ Department of Chemistry, Visva-Bharati, Santiniketan, 731235 West Bengal, India

^2^ Department of Zoology, Visva-Bharati, Santiniketan, 731235 West Bengal, India.

^3^ Department of Chemistry, University of Jyvaskyla, P.O. Box 35, Survontie 9 B, 40014 Jyväskylä, Finland.

**Contents**

1. Materials and Instrumentation...............................................................................................S3
2. NMR Studies.........................................................................................................................S4
3. HRMS Spectrum...................................................................................................................S6
4. Crystallographic Data...........................................................................................................S7
5. UV-Vis and Fluorescence Titration....................................................................................S8
6. Calculation of Binding Constants of **NAF** Towards Colistin.............................................S9
7. Calculation of Limit of Detection (LOD) and Limit of Quantification with Colistin................................................................................................................................S10
8. Job’s Plot for Determining the Stoichiometry of Binding by Fluorescence Method................................................................................................................................S12
9. Competitive fluorescence selectivity studies of NAF with various relevant biomolecules, Metal ions, antibiotics and vitamins:...................................................................................S13
10. pH Titration Curve of **NAF** Upon Addition of Colistin ………………………………….S14
11. Geometry Optimization using Density Functional Theory (DFT) and TDDFT ...……………………………………………………………………………………….…S15
12. Non-covalent Interactions Geometry Optimization using Density Functional Theory (DFT) and TDDFT ....................................................................................................................S19
13. NMR Titration Studies ………...…………………………………………………..…..S20
14. In Vitro Experiment with Zebrafish.......................................................................……..S21
15. Experiment with Poultry Chicken ………………………………………………….....S23

**1.** **Materials and Instrumentation:**

All reagents were purchased from Sigma-Aldrich. Unless otherwise mentioned, materials were obtained from commercial suppliers and were used without further purification. Solvents were dried according to standard procedures. Elix Millipore water was used throughout all experiments. ^1^H- and ^13^C NMR spectra were collected at 400 and 100 MHz, respectively, on a Bruker DRX spectrometer. For NMR spectra, DMSO-*d*_6_ and for NMR titration DMSO-*d*_6_ and D_2_O were used as solvent using TMS as an internal standard. Chemical shifts are expressed in δ ppm units and ^1^H–^1^H coupling constants in Hz. The following abbreviations are used to describe spinmultiplicities in ^1^H NMR spectra: s = singlet; d = doublet; t = triplet; m = multiplet. Thin Layer Chromatography (TLC) was performed using silica gel 60 F_254_ (Merck) plates. Fluorescence spectra were recorded on a Perkin Elmer Model LS 55 spectrophotometer. UV spectra were recorded on a SHIMADZU UV-3101PC spectrophotometer.

Leibovitz-15 medium, foetal bovine serum, collagenase were purchased from Himedia Laboratories. Hank’s solution, HEPES were obtained from Sigma-Aldrich. Colistin sulfate was procured from SRL.

**NMR data of NAF**

^1^H NMR (400 MHz, DMSO-*d*_6_): δ (ppm) = 13.07 (s, 1H), 9.76 (s, 1H), 8.68 (s, 1H), 8.51-8.49 (d, 1H, J = 8 Hz), 8.00-7.88 (dd, 2H, J = 48 Hz), 7.59-7.55 (m, 1H, J = 16 Hz), 7.43-7.39 (m, 1H, J = 16 Hz), 7.25-7.22 (d, 1H, J = 12 Hz), 7.12-7.11 (d, 1H, J = 4 Hz), 6.57-6.56 (d, 1H, J = 4 Hz), 5.48 (s, 1H) 4.52-4.51 (d, 2H, J = 4Hz). ^13^C NMR (400 MHz, DMSO-d_6_): δ (ppm) = 161.04, 160.11, 159.80, 150.31, 148.10, 132.20, 128.89, 128.03, 127.76, 123.75, 121.34, 119.42, 118.77, 109.74, 108.34, 55.90. HRMS (TOF MS): (m/z, %): Calcd. for C_17_H_14_N_2_O: 294.10. Found: m/z = 317.0048.

**2. NMR Studies**

**^1^H NMR of NAF in DMSO-*d_6_*:**

**
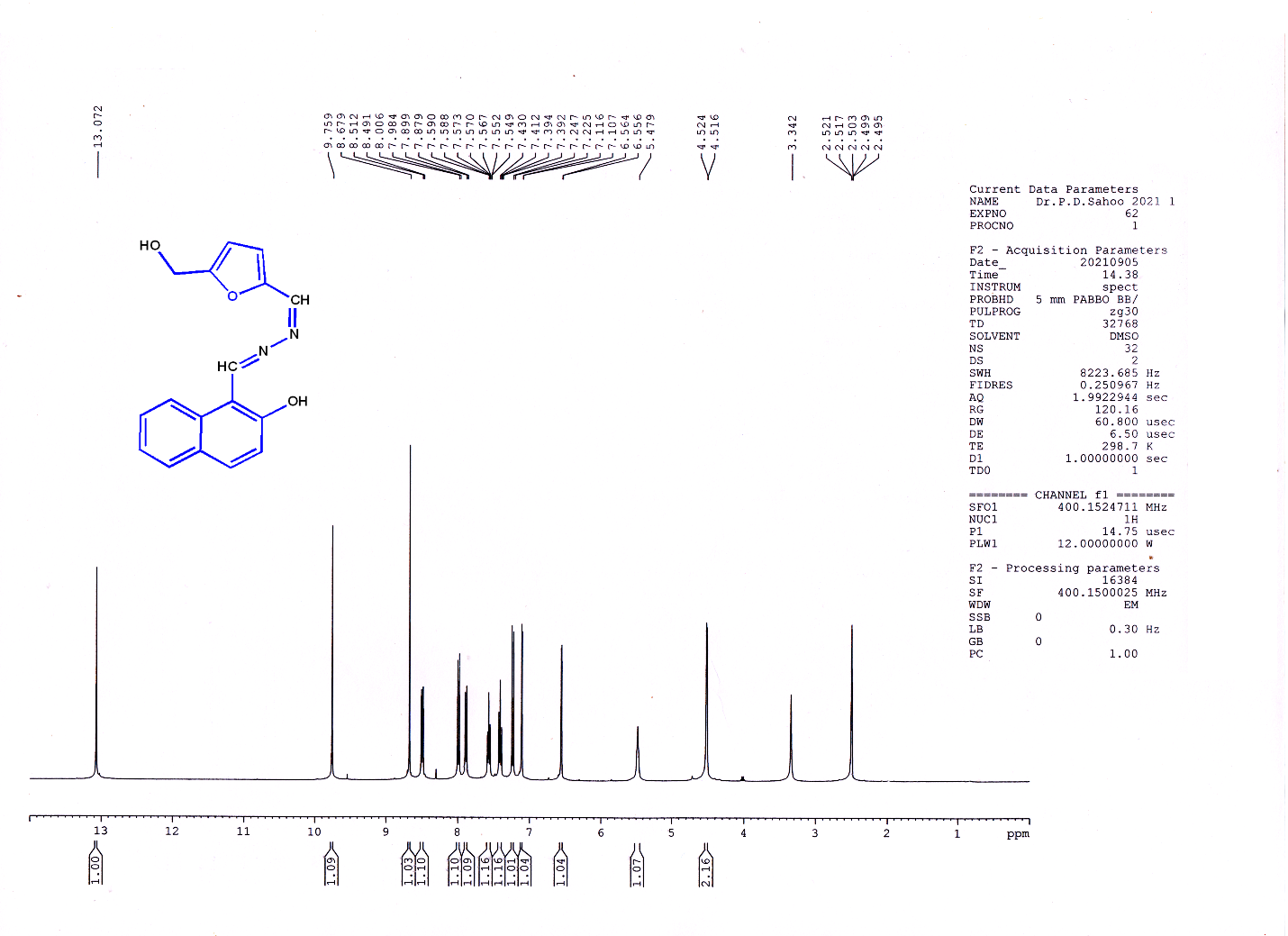
**

**Figure S1.** **^1^**H NMR of **NAF** in DMSO-*d*_6_ (400 MHz, 298 K).

**^13^C NMR of NAF in DMSO-*d_6_*:**

**
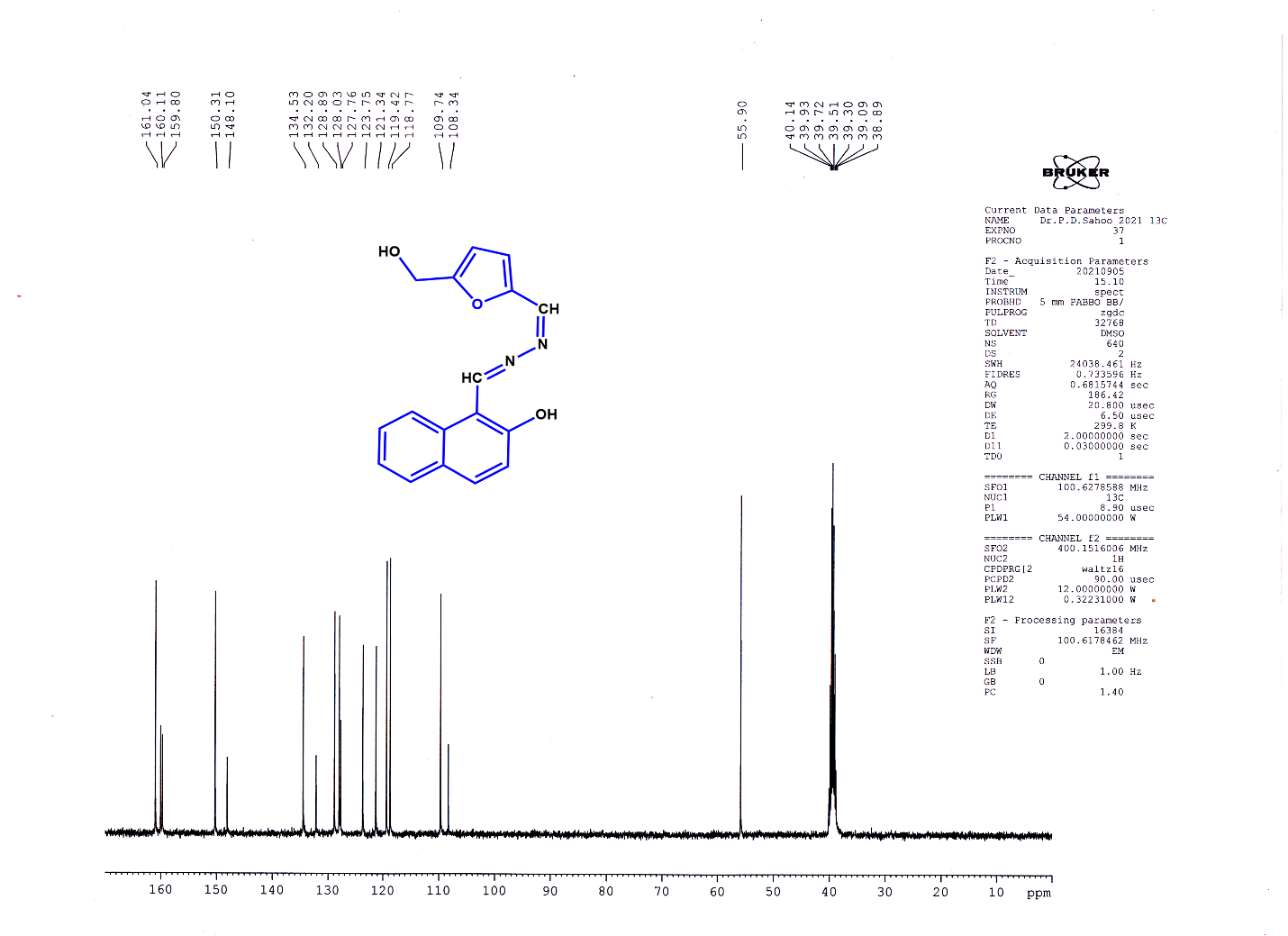
**

**Figure S2.** **^13^**C NMR of **NAF** in DMSO-*d*_6_ (100 MHz, 300 K).

**3. HRMS of NAF**

**
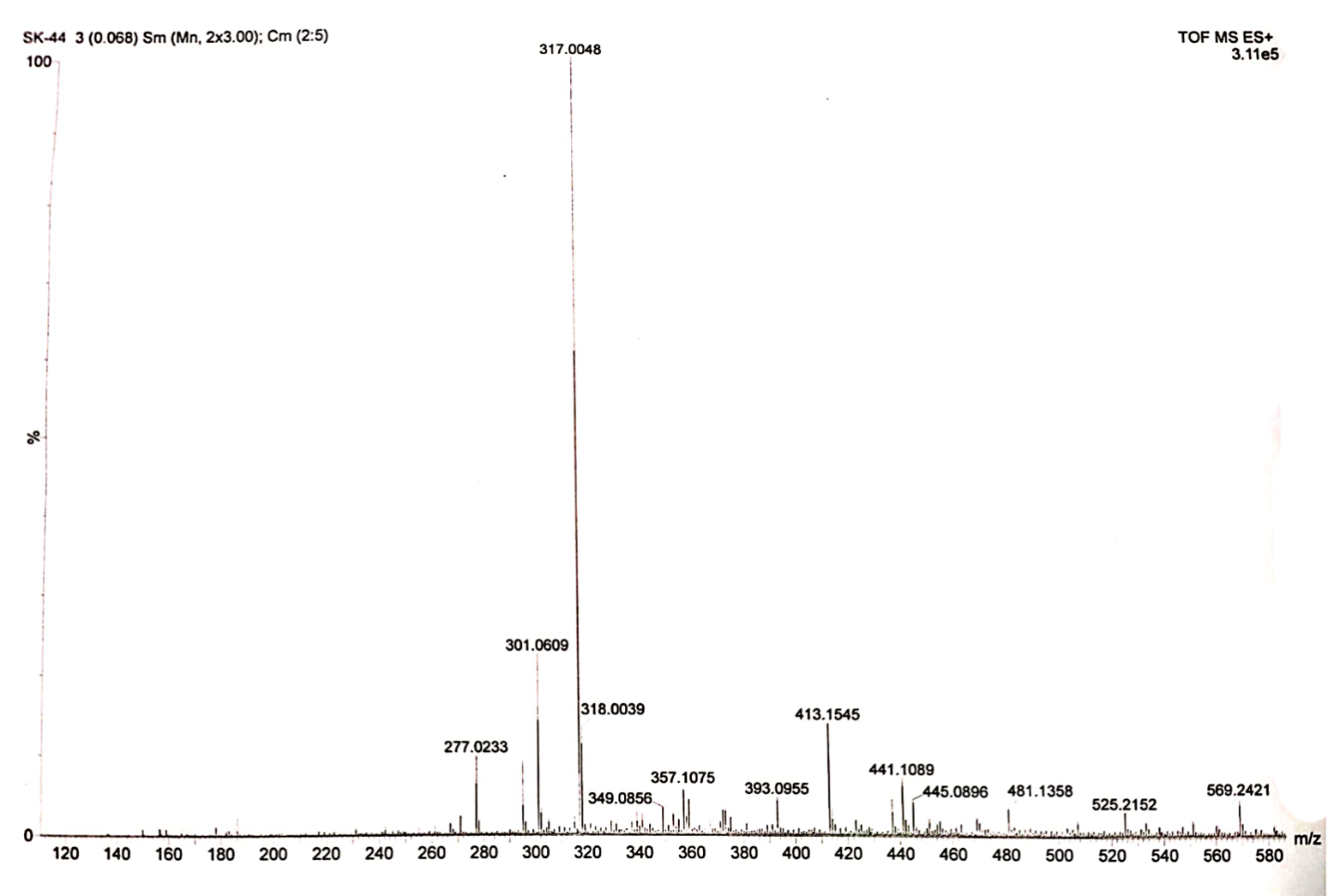
**

**Figure S3.** HRMS of **NAF**.

**4. Crystallographic Data**

**^
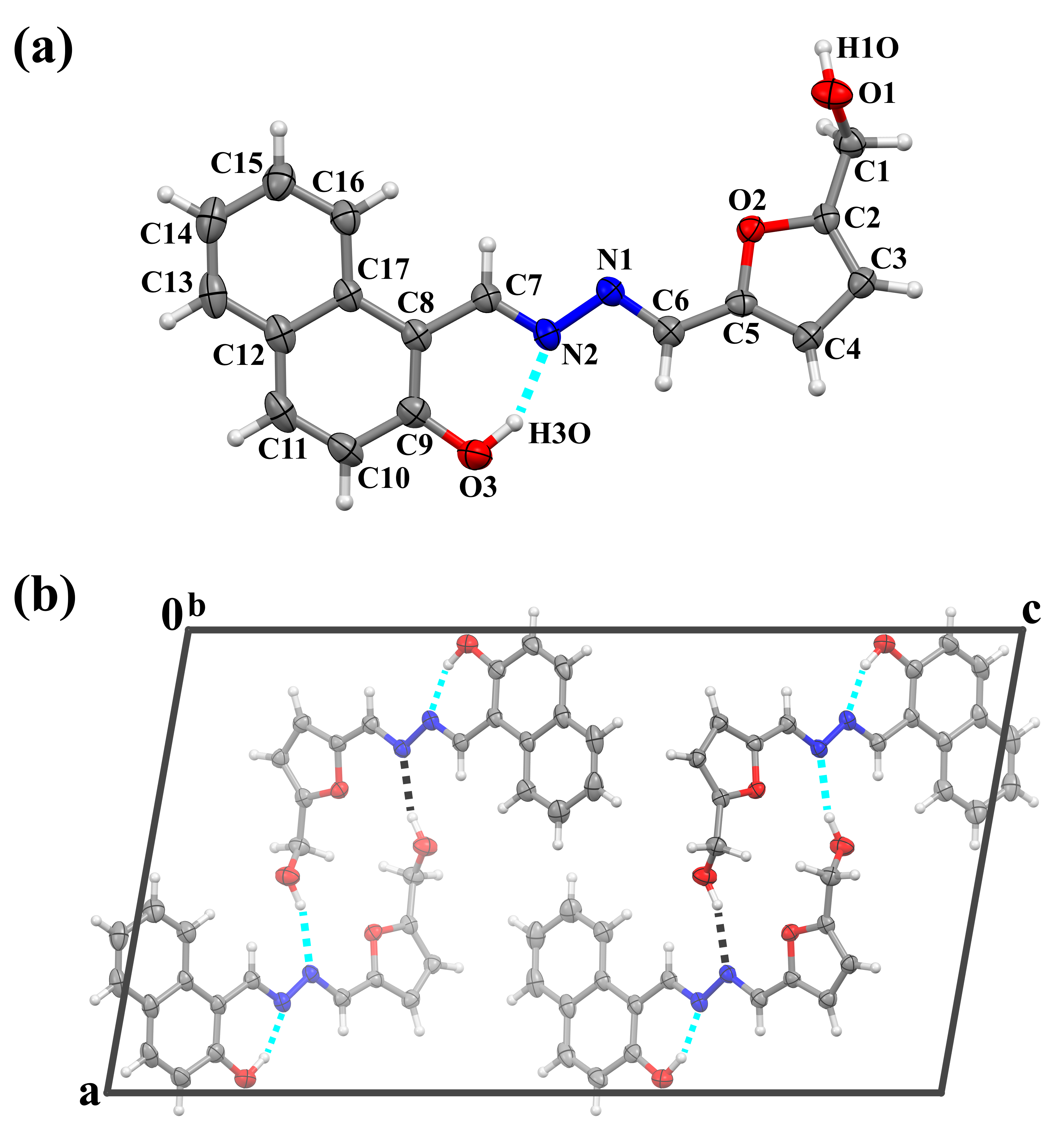
^**

**Figure S4.** (a) Displacement ellipsoid plot of **NAF** and (b) its crystal packing. Displacement ellipsoids are drawn at 50% probability level. Selected bond distances (Å) and angle (°): O1−H1O 0.92, N1^a…^H1O 2.022, O1^…^N1^a^ 2.903, O1−H1O^…^N1^a^ 158.1, O3−H3O 0.92, N2^…^H3O 1.709, O3^…^N2 2.543, O3−H3O^…^N2 148.5. Symmetry operator: a = 1−*x*, 0.5+*y*, 0.5−*z*.

**5. UV-Vis and Fluorescence Titration**

Absorption and fluorescence titration spectra of **NAF** upon increamental addion of colistin.


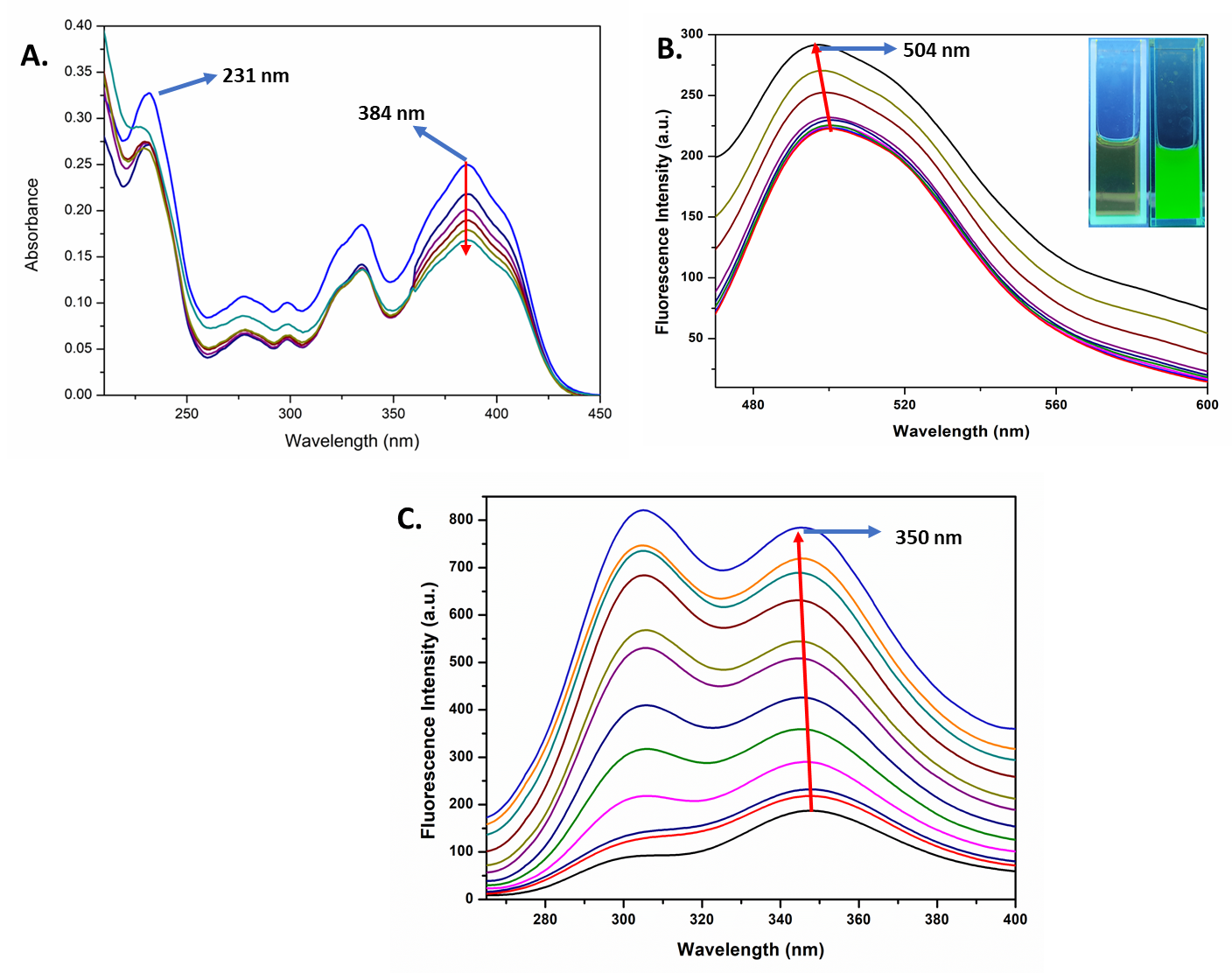


**Figure S5.** (A) UV−Vis absorption spectra of **NAF** (10 μM) upon incremental addition of colistin up to 3.85 µM in CH_3_CN:H_2_O (2:5, v/v) at pH 7.0 (10 mM phosphate buffer); (B) Fluorescence emission spectra at 504 nm (λ_ex_ = 384 nm) of **NAF** (10 μM) at varied concentrations of colistin (upto 3.84 µM) in CH_3_CN:H_2_O (2:5, v/v) at pH 7.0 (10 mM phosphate buffer) [Inset: fluorimetric change of **NAF** upon interaction with colistin]. (C) Fluorescence emission spectra at 350 nm (λ_ex_ = 231 nm) of **NAF** (10 μM) at varied concentrations of colistin (upto 3.84 µM) in CH_3_CN:H_2_O (2:5, v/v) at pH 7.0 (10 mM phosphate buffer) ( filter open , slit 10/10)

**6. Calculation of Binding Constants of NAF Towards Colistin**

**

**

**Figure S6.** Linear regression analysis for the calculation of binding constant values **NAF** towards colistin.

The association constant (K_a_) of **NAF** for colistin has been determined from the equation: K_a_ = intercept/slope.

From the linear fit graph we get intercept = 0.26799, slope = 5.777X10^-7^.

Thus we get, **K_a_** = (0.26799)/(5.777X10^-7^)**= 0.04638x10^7^ M^-1^ =** **4.638x10^5^ M^-1^**

**7.** **Calculation of Limit of Detection (LOD) and Limit of Quantification with Colistin**

The detection limit of the chemosensor **NAF** for colistin has been calculated on the basis of fluorescence titration. To determine the standard deviation for the fluorescence intensity, the emission intensity host molecule (without colistin) was measured by 5 times and the standard deviation of blank measurements was calculated.

The limit of detection (LOD) of **NAF** for sensing colistin was determined from the following equation^.^

LOD = K × 3*σ*/*m*

where K = 2 or 3 (we take 3 in this case); *σ* is the standard deviation of the blank receptor solution; m corresponds to the slope of the calibration curve.





**Figure S7.** Linear fit curve of **NAF** at 350 nm with respect to colistin concentration. Standard deviations are represented by error bar (n = 3).

**Table S1. Standard deviation for NAF.**

| **Blank Reading** | **Fluorescence Intensity** |
| --- | --- |
| **Reading 1** | 184.34 |
| **Reading 1** | 185.61 |
| **Reading 1** | 183.76 |
| **Reading 1** | 186.58 |
| **Reading 1** | 186.83 |
| **Standard deviation(σ)** | 1.2040664433494 |

| **Standard Deviation (σ)** | **1.204** |
| --- | --- |
| **Slope from Graph (m)** | 2.39346X10^8^ |

Thus, Limit of Detection (**LOD**) = 3*σ*/*m* = (3×1.204)/2.2917x10^8^ =1.576x10^-8^ [M]

**Determination of limit of quantification (LOQ)**

The limit of quantification (**LOQ) =** 10*σ/m =* (10×1.204)/2.2917x10^8^ = 5.253x10^-8^ [M]

**8.** **Job’s Plot for Determining the Stoichiometry of Binding by Fluorescence Method**

**

**

**Figure S8.** Job’s plot of **NAF** (10 µM) with colistin (10 µM) at neutral pH value (pH = 7.0, 10 mM phosphate buffer) by fluorescence method, which indicate 1:1 stoichiometry for **NAF** with colistin. Standard deviations are represented by error bar (n = 3).

**
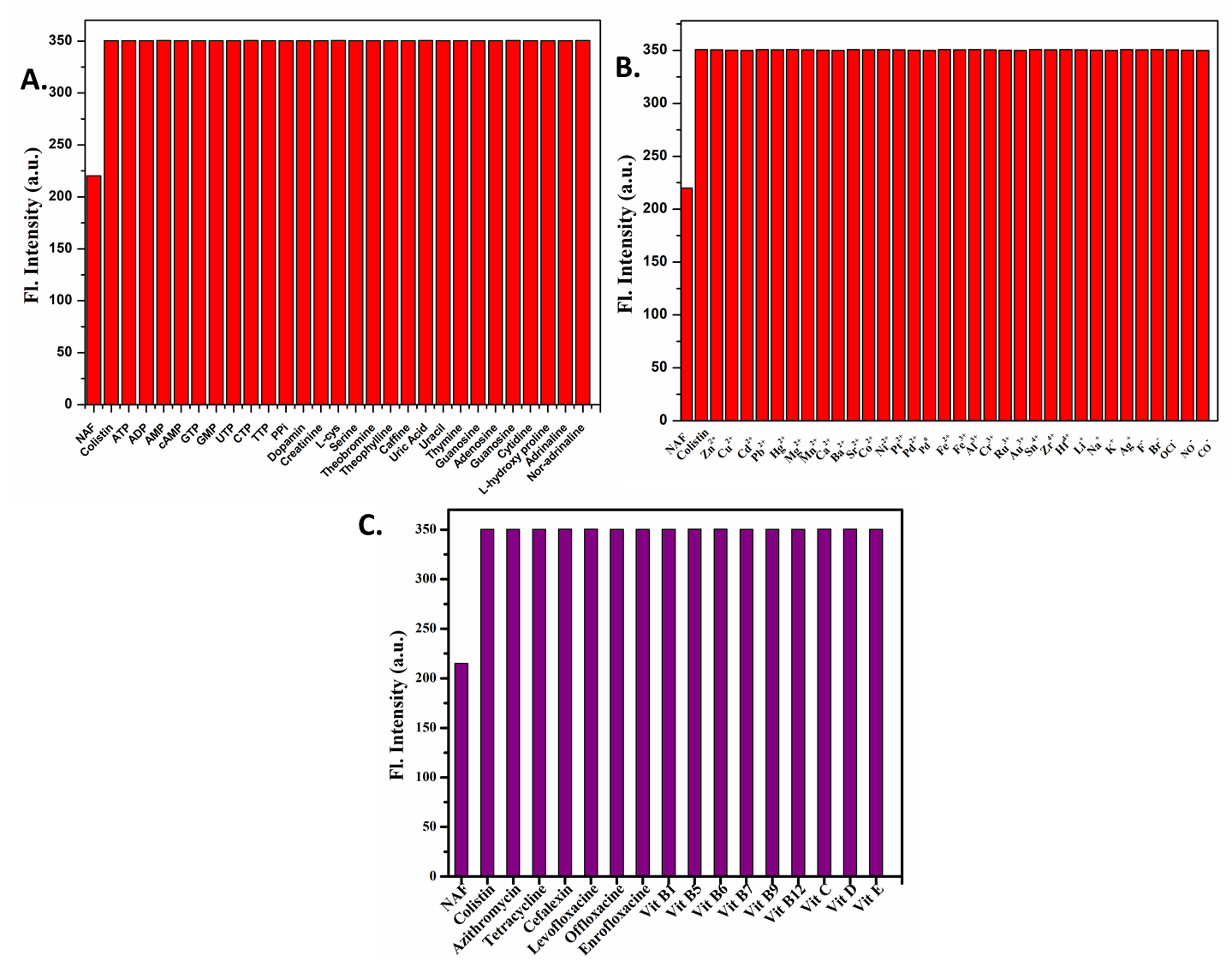
9. Competitive fluorescence selectivity studies of NAF with various relevant biomolecules , Metal ions , antibiotics and vitamins**

**Figure S9.** Histogram representing competitive fluorescence spectra of **NAF** , **NAF**+colistin in presence of other analytes ( relelevant biomolecules, metal ions, antibiotics and vitamins) with various nucleotides and biological phosphates at 504 nm (λ_ex_= 384 nm) in CH_3_CN: H_2_O: (2:5, v/v) at neutral pH (pH=7.0, 10 mM phosphate buffer).

**10. pH Titration Curve of NAF Upon Addition of Colistin**

**

**

**Figure S10.** Effect of pH value on the fluorescence intensity of **NAF** (10μM) in the absence of colistin (100 μM) and in presence of colistin.

**11. Geometry Optimization Using Density Functional Theory (DFT)**

Interaction mechanism of **NAF** with colistin has been investigated by quantum mechanical calculations by employing the DFT at Gaussian09 programme. Solvent effects were incorporated using CPCM solvent model.

Energy optimized geometries of **NAF** and **NAF-colistin** complex obtained at the 6-31G(d,p) levels of theory with CPCM solvation .

**Table S2.** Details of the geometry optimization in Gaussian09 program.

| **Details** | **NAF** | **NAF in its binding mode** | **NAF-colistin** | **colistin** |
| --- | --- | --- | --- | --- |
| **Calculation method** | B3LYP | B3LYP | B3LYP | B3LYP |
| **Basis** | 6-31G(d,p) | 6-31G(d,p) | 6-31G** | 6-31G** |
| **Energy (Kcal/mol)** | -621977.79 | -622075.59 | -3066113.62 | -2444019.18 |
| **Charge, Multiplicity** | 0, 1 | 0, 1 | 0, 1 | 0, 1 |
| **Solvent (CPCM)** | Acetonitrile | Acetonitrile | Acetonitrile | Acetonitrile |

Ground-state energy optimized structures of probe **NAF** and difference in orientation while binding with colistin. NAF structure obtained from crystal structure of the compound and **NAF** in its binding mode corresponds to structure when interacting with colistin.

**
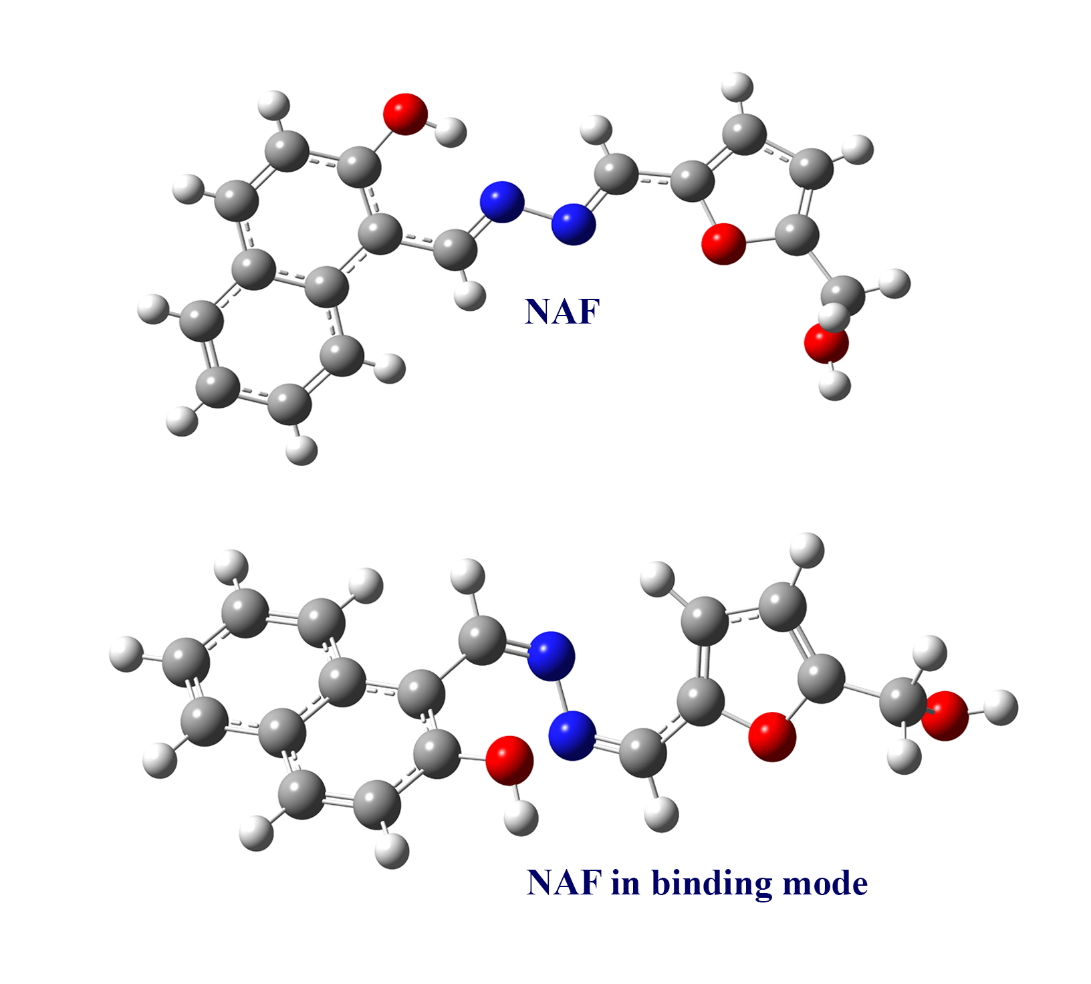
**

**Figure S11.** Energy optimized structures of **NAF** and **NAF** in its binding mode.

**
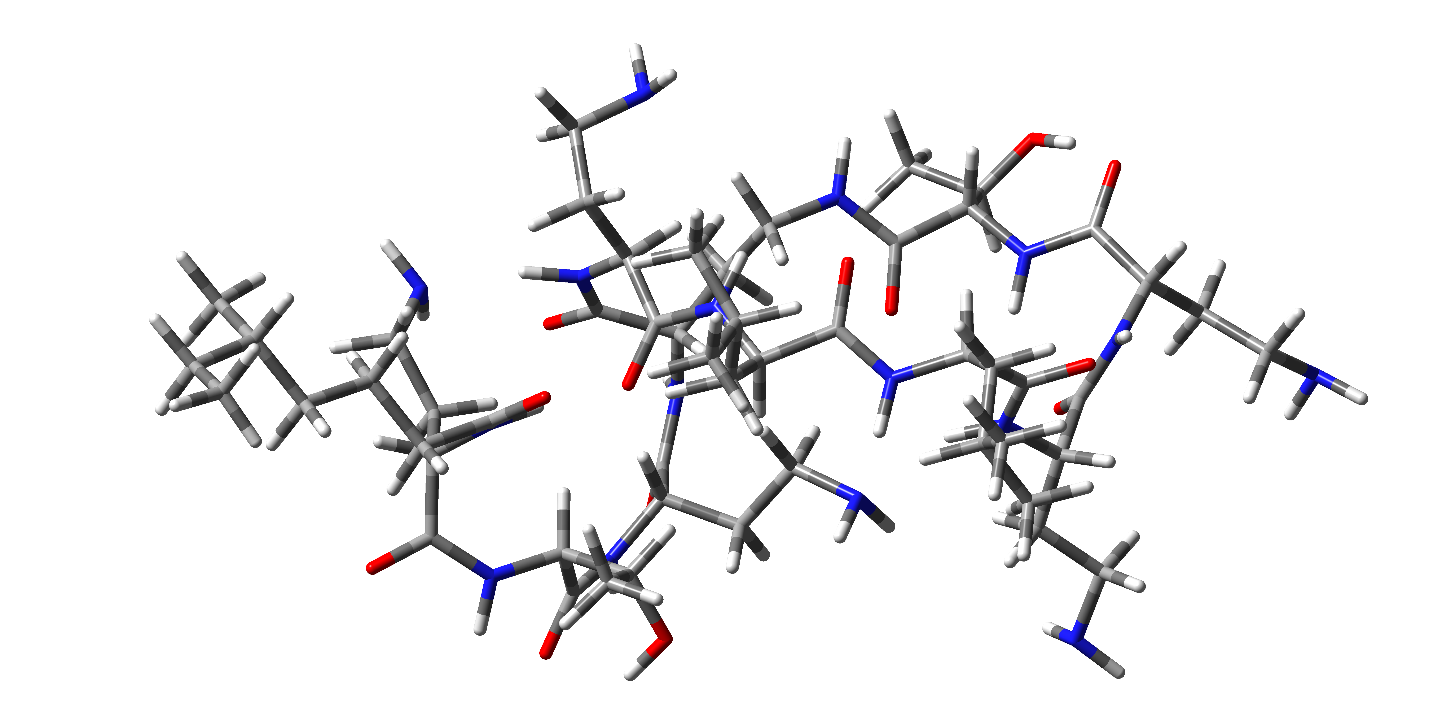
**

**Figure S12.** Ground-state energy optimized geometry of colistin structure.

**Table S3.** Selected electronic excitation energies (eV), oscillator strengths (f), main configurations of the low-lying excited states of all the molecules and complexes. The data were calculated by TDDFT//B3LYP/6-31G(d,p) based on the optimized ground state geometries.

|  | **Functional** | | **Electronic Transition** | | **Molecular orbitals associated with the transitions** | | **Excitation**  **Energy^a^ (eV)** | | **Corresponding Absorption Wavelength (nm)** | | **f^b^** | | **Composition^c^(%)** | |
| --- | --- | --- | --- | --- | --- | --- | --- | --- | --- | --- | --- | --- | --- | --- |
| **NAF** | | **B3LYP** | | S_0_ → S_1_ | | 77 →78 | | 3.1054 | | 399.25 | | 0.8775 | | H →L (70) |
|  |  |  |  | S_0_ → S_11_ | | 75 →80  77 →80 | | 5.2812 | | 234.77 | | 0.2315 | | H-2→L+2(53)  H→L+2(41) |
|  |  |  |  |  | |  | |  | |  | |  | |  |

**
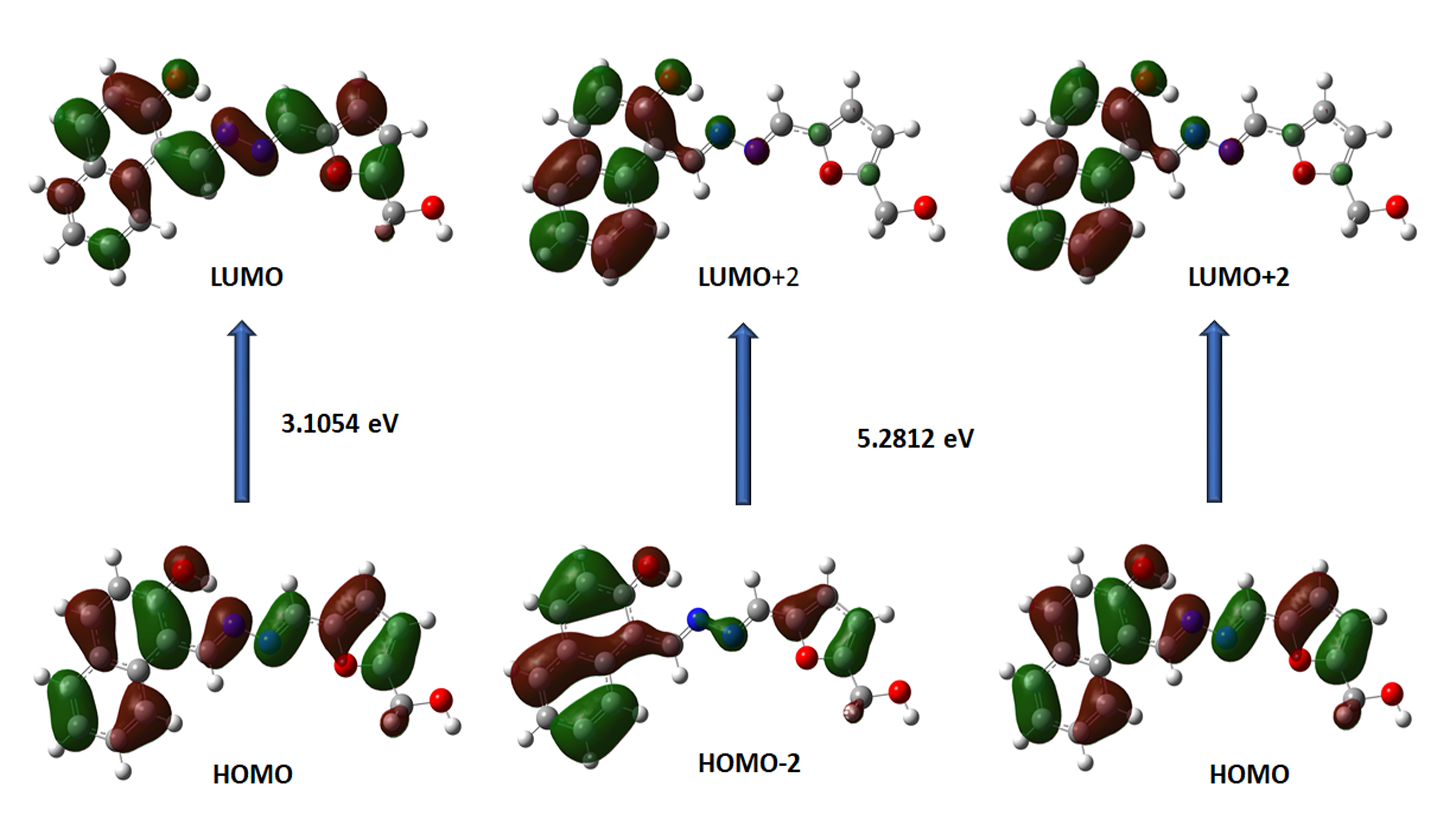
**

**Figure S13.** Frontier molecular orbitals of **NAF** corresponds to the transitions mentioned in the table above.


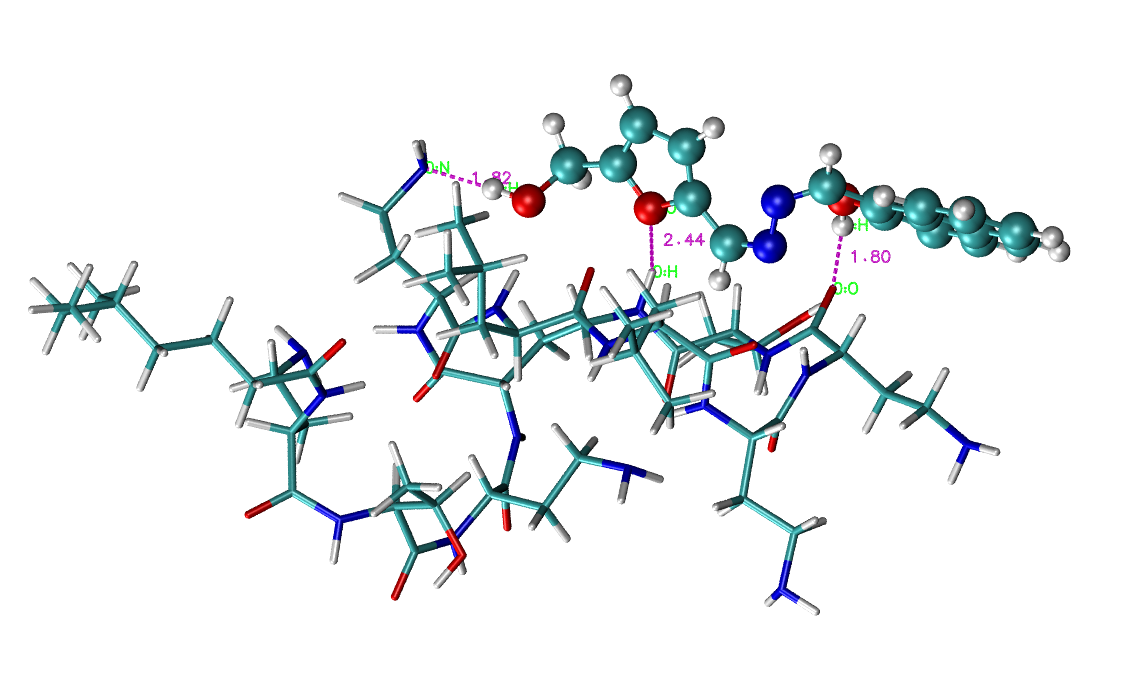


**Figure S14.** Interaction sites of inbetween **NAF** and colistin obtained from quantum mechanical calculations and geometry optimisations

**12.. Non-covalent Interactions**

NCIPLOT has been used to analyse, visualise and establish non-covalent interactions, i.e hydrogen bonding interactions. The non-covalent interactions can be analysed by calculating the inhomogeneous electron distribution at the bond interaction regions. Considering the reduced density gradient ‘s’ and electron density ‘ρ’, it has been found that the reduced gradient is zero at bond critical points.

*s*=[1/^3^√{2(3π)^2^}] x [|∇*ρ*| / *ρ*^4/3^]........(1)

For any weak interaction, a characteristic signal peak is found at 's' versus '*ρ*' plot at low-density low-gradient region. After analysing the Laplacian or the second derivative of the density, one can obtain the following equation

∇^2^ρ = λ_1_+ λ_2_+λ_3,_λ_1_ ≤ λ_2_ ≤ λ_3_ ......(2)

where λ_i_ represents three eigenvalues of the electron-density Hessian matrix which are components of the maximal variation along three-principal axis. The sign of λ_2_ efficiently helps to determine whether the interaction is attractive (λ_2_ < 0) or repulsive (λ_2_ > 0). The strength of the interaction can also be obtained from the density 'ρ'. Therefore plot of 's' versus sin(λ_2_)ρ provides a quantitative molecular interaction index which can be interpreted as qualitative and quantitative measure of non-covalent interactions present in the system.^1^

**
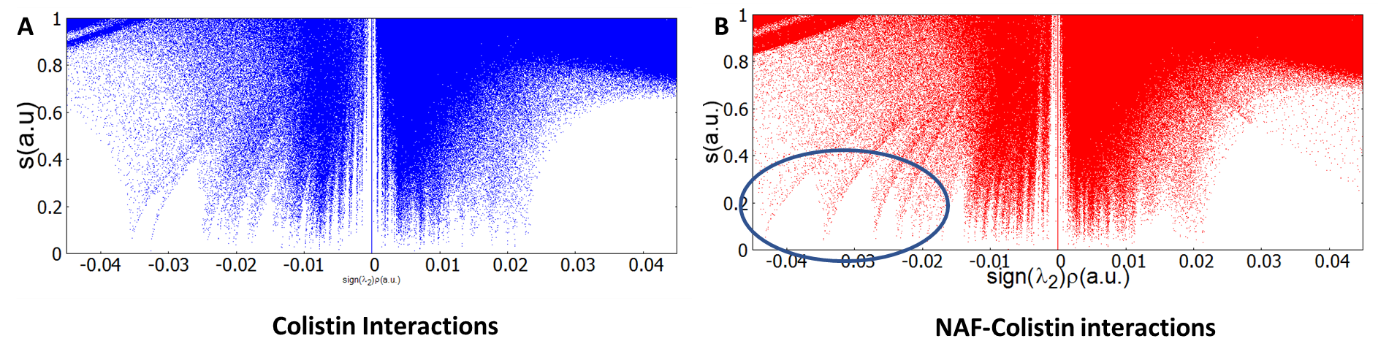
**

**Figure S15.** Plots of reduced density gradient (‘s’) at ‘Y-axis’ and electron density multiplied by the sin of the second Hessian eigenvalue (sin(λ_2_)ρ) at ‘X-axis’ for colistin and **NAF**-colistin.

Figure S15 shows appearance of newly dicreate peaks at low density low gradiant negative region.

**13. NMR Titration Studies**

**
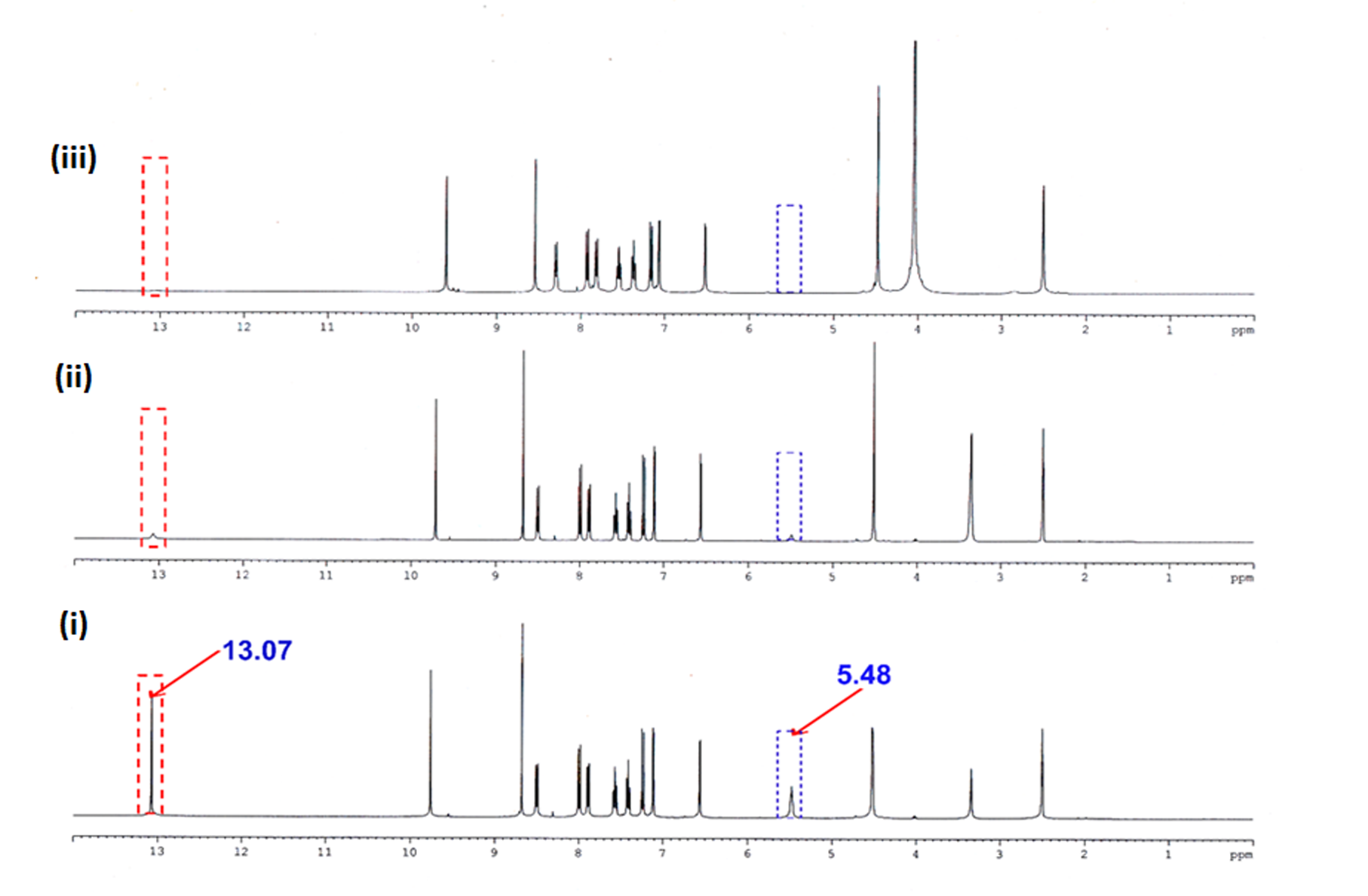
**

**Figure S16.** ^1^H NMR titration [400 MHz] of **NAF** in DMSO-*d*_6_ at 25°C and the corresponding changes after addition of colistin in D_2_O where (i) only **NAF**, (ii) NAF + 65 µL colistin and (iii) **NAF +** 165 µL colistin.

**14. *In Vitro* Experiment with Zebrafish**

**Table S4.** Phothographs of zebrafish experiment.

| Zebrafish | Zebrafish | Zebrafish liver |
| --- | --- | --- |
| 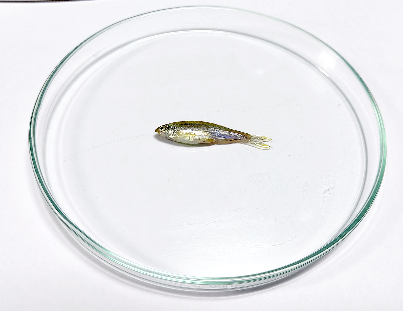 | 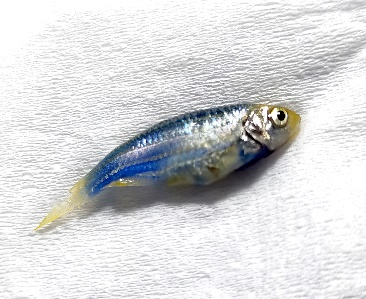 | 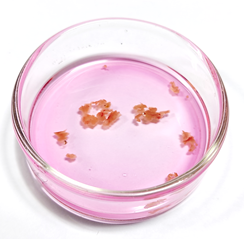 |


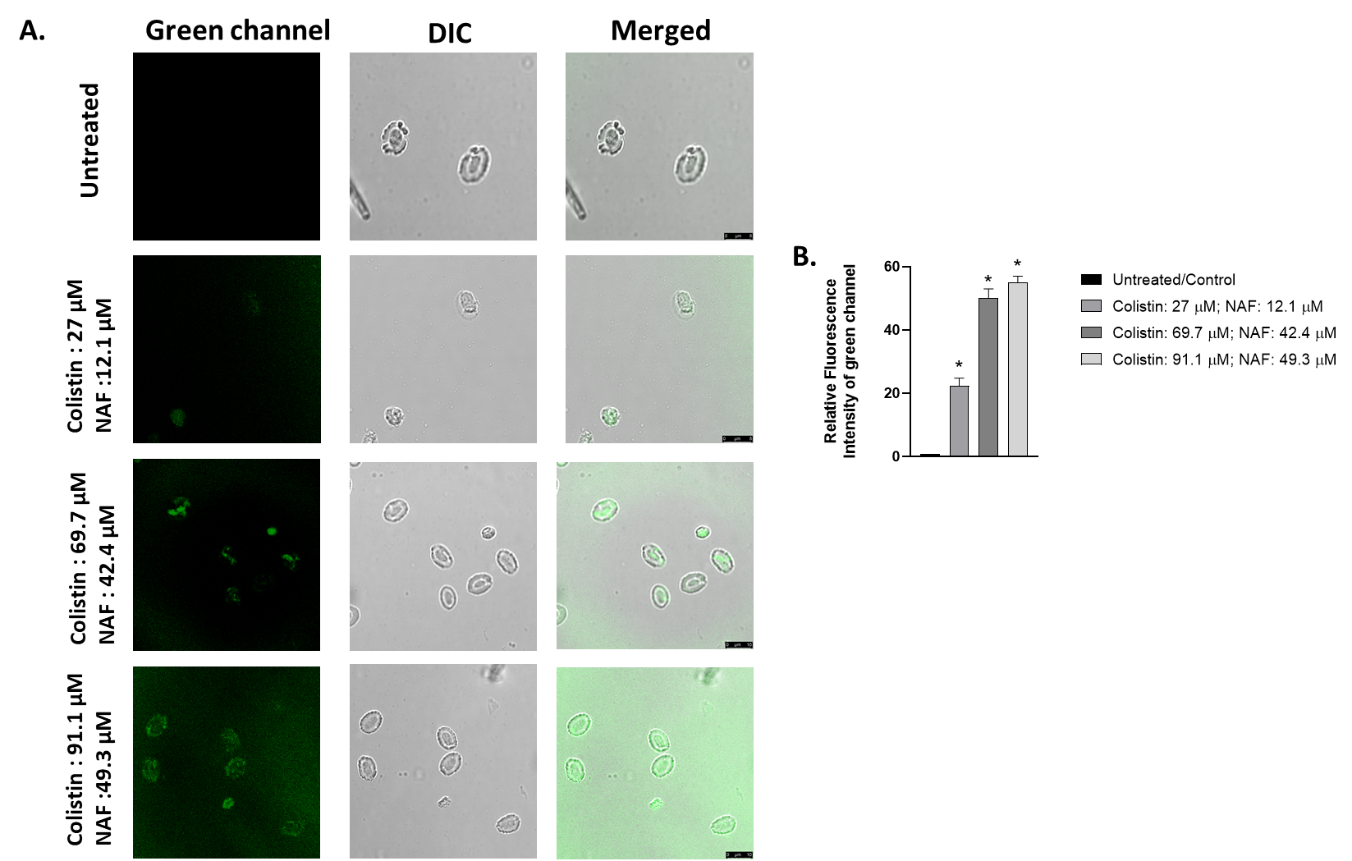


**Figure S17.** Confocal microscopic images of isolated hepatocytes from zebrafish showing dose kinetics from cells (A) that were either left untreated (control) or treated with colistin 27µM, 69.7 µM, 91.1µM and **NAF** 12.1 µM, 42.4 µM, 49.3 µM for 75 and 45 min. Green channel, DIC images and merged images are shown. Scale bar is 8 μm. Zoom factor = 3.5. Magnification = 630x. (B) Relative fluorescence intensity of the above-mentioned images was quantified using ImageJ v 1.46 software and graphed.


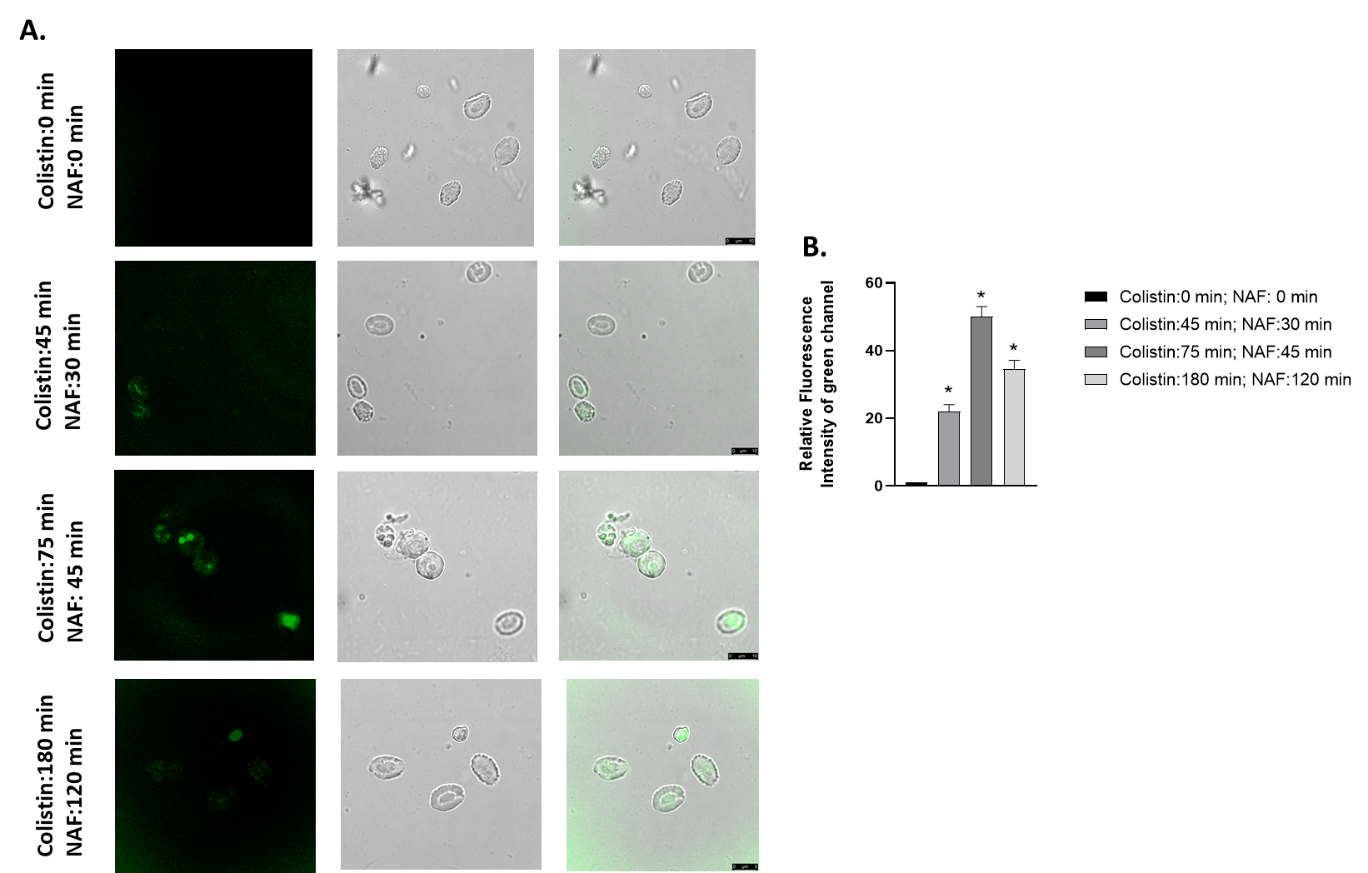


**Figure S18.** Confocal microscopic images of isolated hepatocytes from zebrafish showing time kinetics from cells (A) that were either left untreated (control), 0 min or treated with 69.7 µM colistin and 42.4 µM **NAF** for 45 and 30, 75 and 45, 180 and 120 minutes, respectively. Green channel, DIC images and merged images are shown. Scale bar is 8 μm. Zoom factor = 3.5. Magnification = 630x. (B) Relative fluorescence intensity of the above-mentioned images was quantified using ImageJ v 1.46 software and graphed.

**15. Experiment with Poultry Chicken**

**Table S5.** Details of poultry chicken experiment.

| Control |  | Representative picture of chicken from each set | Weight |
| --- | --- | --- | --- |
|  | Day 1 | 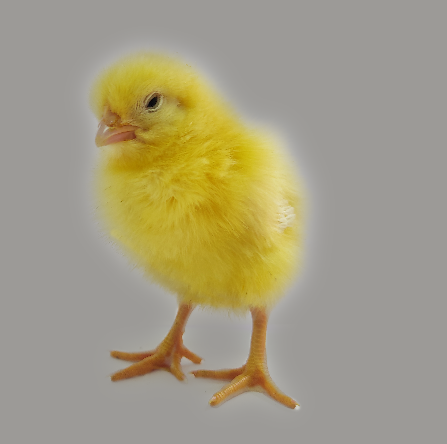 | 32 g |
|  | Day 15 | 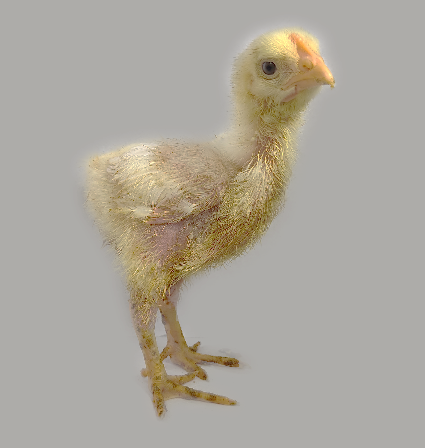 | 154 g |
|  | Day 32 | 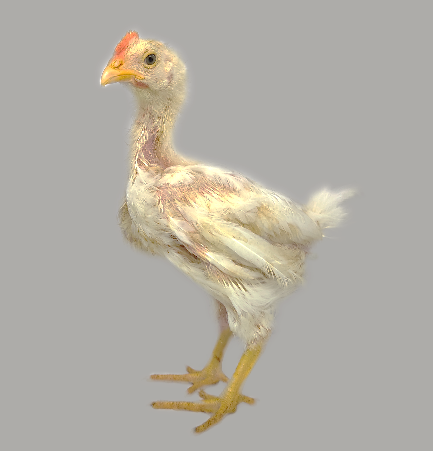 | 496 g |
|  | Day of dissection  Day 36 | 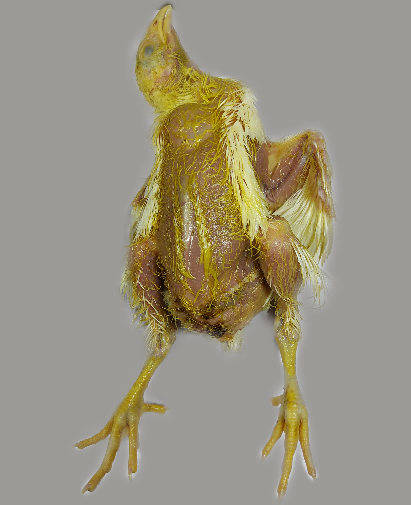 | 525 g |
| Treated | Day 1 | 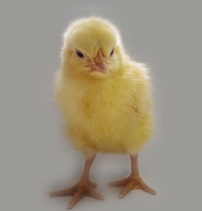 | 36 g |
|  | Day 15 | 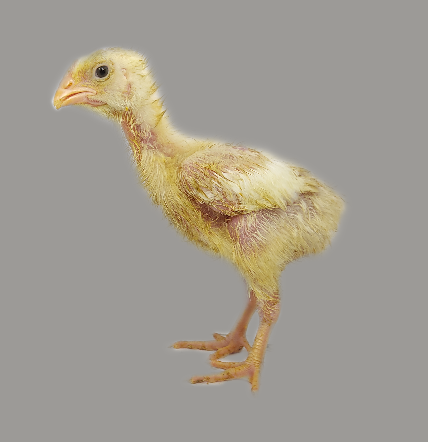 | 209 g |
|  | Day 32 | 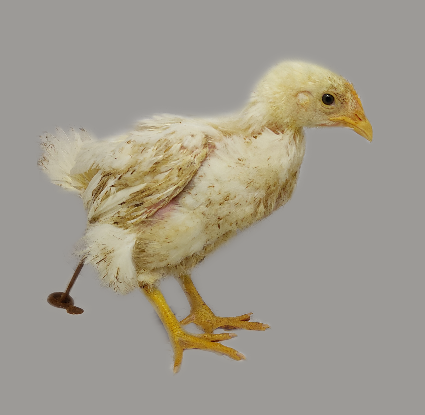 | 664 g |
|  | Day of disection  Day 36 | 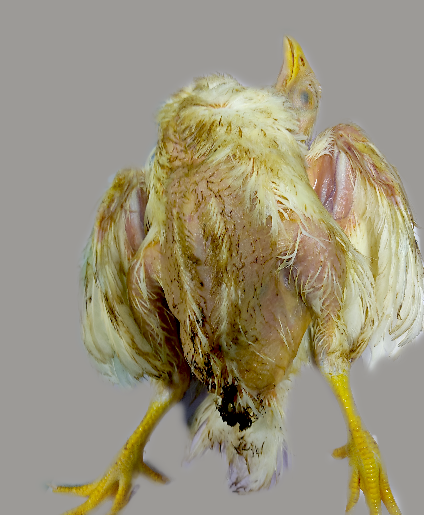 | 675 g |


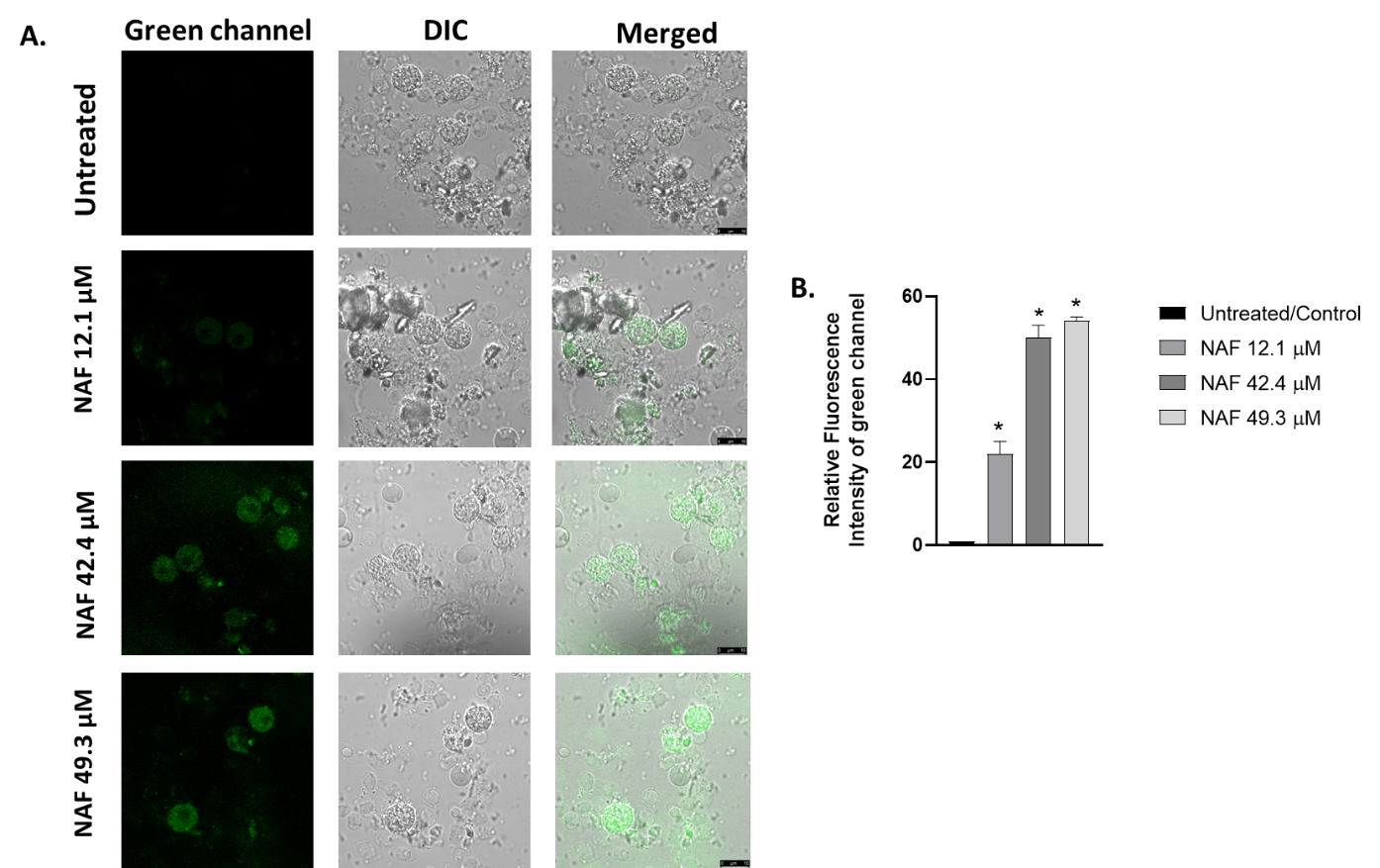


**Figure S19.** Confocal microscopic images of isolated hepatocytes from chicken showing dose kinetics (A) that were either left untreated (control) or treated with **NAF** 12.1, 42.4, 49.3 µM and colistin for 32 days. Green channel, DIC images and merged images are shown. Scale bar is 10 μm. Zoom factor = 2.4. Magnification = 630x. (B) Relative fluorescence intensity of the above-mentioned images was quantified using ImageJ v 1.46 software and graphed.

References:

1. P. Bandyopadhyay, S. Ray, M. Seikh, *Phys. Chem. Chem. Phys.* **2019**, *21* (48), 26580–26590.
